# Bronchial Aspirate-Based Profiling Identifies MicroRNA Signatures Associated With COVID-19 and Fatal Disease in Critically Ill Patients

**DOI:** 10.3389/fmed.2021.756517

**Published:** 2022-02-03

**Authors:** Marta Molinero, Iván D. Benítez, Jessica González, Clara Gort-Paniello, Anna Moncusí-Moix, Fátima Rodríguez-Jara, María C. García-Hidalgo, Gerard Torres, J. J. Vengoechea, Silvia Gómez, Ramón Cabo, Jesús Caballero, Jesús F. Bermejo-Martin, Adrián Ceccato, Laia Fernández-Barat, Ricard Ferrer, Dario Garcia-Gasulla, Rosario Menéndez, Ana Motos, Oscar Peñuelas, Jordi Riera, Antoni Torres, Ferran Barbé, David de Gonzalo-Calvo

**Affiliations:** ^1^Translational Research in Respiratory Medicine, University Hospital Arnau de Vilanova and Santa Maria, IRBLleida, Lleida, Spain; ^2^CIBER of Respiratory Diseases (CIBERES), Institute of Health Carlos III, Madrid, Spain; ^3^Intensive Care Department, University Hospital Arnau de Vilanova, IRBLleida, Lleida, Spain; ^4^Hospital Universitario Río Hortega de Valladolid, Valladolid, Spain; ^5^Group for Biomedical Research in Sepsis (BioSepsis), Instituto de Investigación Biomédica de Salamanca (IBSAL), Salamanca, Spain; ^6^Servei de Pneumologia, Hospital Clinic, Universitat de Barcelona, IDIBAPS, Barcelona, Spain; ^7^Intensive Care Department, Vall d'Hebron Hospital Universitari, SODIR Research Group, Vall d'Hebron Institut de Recerca (VHIR), Barcelona, Spain; ^8^Barcelona Supercomputing Center (BSC), Barcelona, Spain; ^9^Pulmonology Service, University and Polytechnic Hospital La Fe, Valencia, Spain; ^10^Hospital Universitario de Getafe, Madrid, Spain

**Keywords:** acute respiratory distress syndrome, COVID-19, mechanical ventilation, microRNA, SARS-CoV-2

## Abstract

**Background:**

The pathophysiology of COVID-19-related critical illness is not completely understood. Here, we analyzed the microRNA (miRNA) profile of bronchial aspirate (BAS) samples from COVID-19 and non-COVID-19 patients admitted to the ICU to identify prognostic biomarkers of fatal outcomes and to define molecular pathways involved in the disease and adverse events.

**Methods:**

Two patient populations were included (*n* = 89): (i) a study population composed of critically ill COVID-19 and non-COVID-19 patients; (ii) a prospective study cohort composed of COVID-19 survivors and non-survivors among patients assisted by invasive mechanical ventilation (IMV). BAS samples were obtained by bronchoaspiration during the ICU stay. The miRNA profile was analyzed using RT-qPCR. Detailed biomarker and bioinformatics analyses were performed.

**Results:**

The deregulation in five miRNA ratios (miR-122-5p/miR-199a-5p, miR-125a-5p/miR-133a-3p, miR-155-5p/miR-486-5p, miR-214-3p/miR-222-3p, and miR-221-3p/miR-27a-3p) was observed when COVID-19 and non-COVID-19 patients were compared. In addition, five miRNA ratios segregated between ICU survivors and nonsurvivors (miR-1-3p/miR-124-3p, miR-125b-5p/miR-34a-5p, miR-126-3p/miR-16-5p, miR-199a-5p/miR-9-5p, and miR-221-3p/miR-491-5p). Through multivariable analysis, we constructed a miRNA ratio-based prediction model for ICU mortality that optimized the best combination of miRNA ratios (miR-125b-5p/miR-34a-5p, miR-199a-5p/miR-9-5p, and miR-221-3p/miR-491-5p). The model (AUC 0.85) and the miR-199a-5p/miR-9-5p ratio (AUC 0.80) showed an optimal discrimination value and outperformed the best clinical predictor for ICU mortality (days from first symptoms to IMV initiation, AUC 0.73). The survival analysis confirmed the usefulness of the miRNA ratio model and the individual ratio to identify patients at high risk of fatal outcomes following IMV initiation. Functional enrichment analyses identified pathological mechanisms implicated in fibrosis, coagulation, viral infections, immune responses and inflammation.

**Conclusions:**

COVID-19 induces a specific miRNA signature in BAS from critically ill patients. In addition, specific miRNA ratios in BAS samples hold individual and collective potential to improve risk-based patient stratification following IMV initiation in COVID-19-related critical illness. The biological role of the host miRNA profiles may allow a better understanding of the different pathological axes of the disease.

## Introduction

The characteristic feature of patients with severe COVID-19 is the development of acute respiratory distress syndrome (ARDS). Consequently, more than 15% of hospitalized patients are eventually transferred to the intensive care unit (ICU), where 70–90% of these patients receive invasive ventilation and 20–50% present fatal outcomes (1–3). Although mortality has been mainly concentrated in patients admitted to the ICU, especially in those who require invasive mechanical ventilation (IMV), there is a lack of tools for predicting the progression of the disease in critically ill patients (4). The development of prognostic assays reflecting the risk of clinical decompensation will be useful for medical decision-making. This approach will also provide valuable molecular information on the pathological mechanisms linked to the severe clinical courses of COVID-19. In this scenario, the use of relatively novel omic technologies, such as transcriptomics, emerges as an interesting approach for in-depth molecular phenotypic analyses.

MicroRNAs (miRNAs) are non-coding RNAs (ncRNAs) consisting on transcripts of 19–25 nucleotides that regulate gene expression posttranscriptionally (5). MiRNAs are fine-tuning regulators of diverse biological processes, including differentiation, proliferation and migration; and specially, the regulation of homeostasis and the response to stress (6). Experimental evidence has suggested that these small transcripts are critical components in viral infections and the host defense, particularly among RNA viruses (7). MiRNAs are also found in the extracellular environment. The release of miRNAs into the extracellular space can be passive, resulting from tissue damage, or active, which is due to secretion mechanisms (8). Actively secreted miRNAs participate in cell-to-cell communication as signaling molecules by regulating the gene expression of recipient cells (9). In addition, the extracellular forms of miRNAs have been recognized as biomarkers with great potential for patient management. Their concentration and expression profile vary in response to physiological stress or pathological insults (10, 11). MiRNAs are highly stable and can be quantified in accessible specimens with techniques currently used in clinical laboratories, i.e., quantitative PCR (qPCR). The role as clinical indicators of miRNAs quantified in a wide array of bodily fluids has been reported (12–14). Although the incorporation of miRNA-based biomarkers to standard clinical care must address several methodological limitations (15), a great progress has been made in the past decade. Some cost-effective tests are currently available in the market to assist in the management of malignant diseases and osteoporosis (16, 17).

While the ICU patient is assisted by IMV, tracheobronchial toilet is crucial to treat the airways obstructed by secretions causing lung atelectasis and the consequent gas exchange deterioration. The specimen extracted by flexible bronchoscopy is known as bronchial aspirate (BAS). BAS is considered a sample type of great interest at the research and clinical levels since it can reflect pathophysiological processes in the local lung environment (18). Furthermore, BAS sampling is a component of standard care, and its collection is less invasive than that of other respiratory specimens, such as bronchoalveolar lavage fluid (BALF).

We hypothesized that critical life-threatening infections induce characteristic molecular changes that can be detected in BAS samples. We first aimed to compare the miRNA profile of BAS samples from critically ill COVID-19 and non-COVID-19 patients to identify molecular pathways associated with the disease. Then, we analyzed the expression profile of miRNAs in BAS samples from survivors and non-survivors of ICU stays. In this second objective, we sought to identify miRNA signatures as prognostic biomarkers of a fatal outcome in COVID-19-related critical illness and to define molecular pathways involved in the development of adverse events.

## Methods

### Study Design and Patients

#### Study 1. BAS miRNA Profiling of Critically Ill COVID-19 and Non-COVID-19 Patients

Eighteen COVID-19-positive patients confirmed by nasopharyngeal swab PCR and 14 non-COVID-19 patients admitted to the ICU from April 2020 to August 2020 were included. Both study groups were matched by age. Comprehensive clinical data were manually extracted from the electronic medical records by specialized clinical research assistants.

The samples and data from patients included were provided by Biobank IdISBa B.0000527 (www.idisba.es) and CIBERES Pulmonary Biobank Consortium B.0000471, a network currently formed by twelve tertiary Spanish hospitals (www.ciberes.org) integrated in the Spanish National Biobanks Network. Samples were processed following standard operating procedures with the appropriate approval of the Ethics and Scientific Committees (s007-BBCOV) and with the collaboration of the healthcare services of the Hospital Universitario Son Espases and Hospital Son Llatzer (Palma, Spain).

#### Study 2. BAS miRNA Profiling of Survivors and Non-survivors to COVID-19-Related Critical Illness

This was a prospective, observational, single-center cohort study. The cohort was composed of consecutive patients 18 years or older with a positive nasopharyngeal swab RT-qPCR test result for SARS-CoV-2 diagnosed with ARDS secondary to COVID-19 infection and under IMV by orotracheal intubation or tracheostomy hospitalized in the ICU from the Hospital Arnau de Vilanova (Lleida, Spain) from March 2020 to January 2021. Demographic, clinical and pharmacological data and information regarding hospital admission, ICU admission, initiation of IMV and outcomes were extracted from the medical records by specialized clinical research assistants. The clinical endpoint of the study was ICU mortality, days under IMV, duration of ICU stay and duration of hospital stay.

Samples were obtained with support by IRBLleida Biobank (B.0000682) and “Plataforma Biobancos PT17/0015/0027.” The procedure to obtain the samples was approved by the Research Ethics Committee (CEIC 2273) and was performed in compliance with the ethical requirements according to the Declaration of Helsinki. The participating patients or their relatives were informed about the research and gave their written informed consent before the use of their biological samples and clinical information in the study.

### Bronchial Aspirates Collection

BAS samples were obtained during the ICU stay as part of the clinical management with two objectives: i) to perform toilet bronchoscopy to aspirate retained secretions and to prevent or to revert lung atelectasis and ii) to rule out superinfections. Bronchoaspiration was performed by flexible bronchoscopy with an Ambu® aScope^TM^ 4 Broncho Large ^5.8/2.8^ bronchoscope (Ambu, Ballerup, Denmark) connected to a vacuum. Patients undergoing this procedure were under continuous sedoanalgesia; in addition, extra boluses of short-acting sedatives (midazolam 5–10 mg) and analgesics (fentanyl 0.05–0.15 mcg) were required to ensure proper tolerance. Bronchial secretion samples (2–5 mL) were obtained. In some cases, simple bronchial washing with 0.9% saline solution (Braun, Melsungen, Germany) was required (19). The samples were immediately aliquoted, frozen and stored at −80°C. For study population 1, the frozen aliquots were shipped on dry ice to the Lleida Institute for Biomedical Research (Lleida, Spain). For study population 2, the samples were stored at the biobank of the Lleida Institute for Biomedical Research.

### RNA Isolation

RNA isolation and miRNA quantification were performed by experienced staff in a blinded fashion. All experiments were performed under standardized conditions in the same laboratory.

A total of 39 miRNAs were selected according to preset criteria by experienced researchers after a comprehensive literature search (Supplementary Table 1). MiRNAs have been previously described as biomarkers of and/or mediators in molecular pathways related to respiratory viral infection, lung damage/fibrosis, immune response, inflammation and coagulation.

BAS samples were thawed at 4°C. RNA isolation was performed in aliquots that had undergone their first thaw. Total RNA was isolated from 100 μL of BAS using the miRNeasy Mini Kit (Qiagen, Hilden, Germany). Five volumes of QIAzol lysis reagent was mixed with one volume of BAS and incubated for 5 min at room temperature. The RNA Spike-In Kit (synthetic UniSp2, UniSp4, and UniSp5) (Qiagen) and *Caenorhabditis elegans* cel-miR-39-3p (1.6 × 10^8^ copies/μL) (Qiagen) were added to all extractions to monitor RNA isolation efficiency. The mixture was supplemented with 1 μg of MS2 bacteriophage RNA (Roche, Merck, Darmstadt, Germany), an RNA carrier not containing miRNAs, to improve RNA yield. All reagents were spiked into samples during RNA isolation after incubation with the denaturing solution. Subsequently, one volume of chloroform was added, and after 3 min at room temperature, the mixture was centrifuged at 12,000 g and 4°C for 15 min. The upper aqueous phase was transferred to a fresh reagent tube, and 1.5 volumes of 100% ethanol were added. Purification of RNA was performed with RNeasy MinElute spin columns according to the manufacturer's instructions. RNA was eluted in 30 μL RNase-free H_2_O and stored in a −80°C freezer.

### RT-qPCR

For isolated total RNA, retrotranscription (RT) was performed using the miRCURY® LNA® RT Kit (Qiagen) according to the manufacturer's instructions. One microliter of RNA was reverse transcribed in 10 μL reactions. Additional spike-in (synthetic UniSp6) (Qiagen) was added to the cDNA synthesis reaction to check for RT robustness and PCR inhibitors. RT reaction was performed with the following conditions: incubation for 60 min at 42°C, heat inactivation for 5 min at 95°C, and immediate cooling to 4°C. Then, cDNA was stored at −20°C.

miRNAs were quantified by qPCR using the miRCURY LNA SYBR® Green PCR Kit (Qiagen) in 384-well miRCURY LNA miRNA Custom PCR Panels (Qiagen) with precoated primers, according to the manufacturer's instructions. This technology is sensitive and specific for miRNA quantification in samples with low RNA yield and allows to discriminate between the target and other closely related mature miRNAs or miRNA precursors. The system provides optimal reproducibility and analytical accuracy (20). cDNA was diluted 40x, and 4 μL were used in 10 μL qPCR reactions. Synthetic UniSp3 was included as an interplate calibrator and qPCR control. The qPCR was performed using a QuantStudio™ 7 Flex Real-Time PCR System under the following conditions: 2 min at 95°C, 40 cycles of 10 s at 95°C and 60 s at 56°C and, finally, melting curve analysis.

### Expression Analysis

QuantStudio Software v1.3 (Thermo Fisher Scientific, Massachusetts, USA) was used for both the determination of the quantification cycle (Cq) and the melting curve analysis. Cq was defined as the fractional cycle number at which the fluorescence exceeded a given threshold. To ensure the highest data quality, RNA and cDNA spike-in levels were analyzed during miRNA quantification. The synthetic spike-in UniSp3 was also used as an interplate calibrator. The miRNAs were considered to be expressed when Cq values < 35. Samples that were below the limit of detection were censored at the lowest level observed for each miRNA. The miRNA ratios were used to reduce sample-to-sample variation in miRNA expression levels. This approach minimizes technical variability caused by sample characteristics, collection and processing (21).

### Pathway and Gene Ontology Enrichment Analyses

Pathway and Gene Ontology (GO) analyses were performed by using the web-based computational tool DIANA-miRPath v3.0 (22) (April 2021). DIANA-miRPath v3.0 utilizes experimentally validated miRNA interactions derived from TarBase v7.0 and combines the results with KEGG (Kyoto Encyclopedia of Genes and Genomes) and GO analyses (biological process). The level of significance was set at a false discovery rate (FDR)-adjusted *p*-value < 0.001.

### Statistical Analysis

The statistical analysis was performed using R software, version 4.0.2 (www.r-project.org). Descriptive statistics were used to summarize the characteristics of the study populations. The normality of the distribution was analyzed using the Shapiro–Wilk test. Data are presented as the mean (standard deviation) or median [25th percentile; 75th percentile] for continuous variables, according to normality, and as the frequency (percentage) for categorical variables. Continuous variables and categorical variables were compared between groups using Student's *t*-test, the Mann–Whitney U test and Fisher's exact test, respectively. Spearman's rho coefficient was used to assess the correlation between continuous variables. The miRNA ratios were calculated using the expression levels: ratio_A/B_ = 2^−*CqA*^ / 2^−*CqB*^. All possible miRNA ratios were generated. The selection of ratios for subsequent statistical analysis was based on the following criteria: (1) higher area under the receiver operating characteristic (ROC) curve (AUC); (2) *p*-value < 0.05; and (3) the same miRNA could not be part of different ratios selected for the statistical analysis. Differences in miRNA ratios between groups were evaluated using linear models for arrays (23). The same models were used to adjust miRNA ratio levels for age and sex. The principal component analysis (PCA) and hierarchical clustering included the differentially expressed miRNA ratios.

A process of miRNA ratio selection for the prediction of mortality based on a random forest algorithm was performed (24, 25). Finally, a predictive model (miRNA ratio score) with the selected miRNA ratios was fitted using a logistic regression model. ROC curves and precision-recall curves were constructed to test the ability of miRNA ratios and clinical predictors to distinguish between survivors and non-survivors. The AUC was used as the global discrimination value measure. For selected features, a miRNA ratio cutoff point was established to fit mortality risk using a maximally selected log-rank statistic (26). Finally, independent models of mortality risk were constructed with a Cox regression model including dichotomized levels of the miRNA ratios. The same analysis was performed with the predictive model. Kaplan-Meier curves were used to illustrate differences among groups in the observed time-to-event outcome. The *p*-value threshold defining significance was set at <0.05.

## Results

### COVID-19 Is Associated With a Specific MicroRNA Profile in Bronchial Aspirates From Critically Ill Patients

We first analyzed the impact of COVID-19 on the miRNA profile in the BAS samples from critically ill patients. The main demographic, clinical, pharmacological and biochemical data of the study groups are summarized in Table 1. The causes of ICU admission among non-COVID-19 patients are displayed in Supplementary Table 2. Compared to non-COVID-19 patients, COVID-19 patients showed a significantly lower PaO_2_ at the time of ICU admission. The duration of the ICU stay, the requirement for non-invasive MV and prone positioning and the use of antibiotics and hydroxychloroquine were also greater in COVID-19 patients.

**Table 1:** Characteristics of critically ill COVID-19 and non-COVID-19 patients (Study Population 1).

	**ALL**	**Non-COVID-19**	**COVID-19**	***p*-value**	**Available data**
	***n* = 32**	***n* = 14**	***n* = 18**		
**Sociodemographic characteristics**					
Age (years), median [P25; P75]	68.0 [62.5; 73.2]	66.5 [64.5; 72.5]	69.5 [59.5; 73.2]	0.939	32
Male, *n* (%)	24 (75.0)	8 (57.1)	16 (88.9)	0.096	32
Smoking history, *n* (%)				0.451	32
Former	9 (28.1)	4 (28.6)	5 (27.8)		
Non-smoker	17 (53.1)	6 (42.9)	11 (61.1)		
Current	6 (18.8)	4 (28.6)	2 (11.1)		
Alcoholism, *n* (%)	3 (9.38)	3 (21.4)	0 (0.0)	0.073	32
**Comorbidities**					
Hypertension, *n* (%)	17 (53.1)	7 (50.0)	10 (55.6)	1.000	32
Type II Diabetes Mellitus, *n* (%)	9 (28.1)	3 (21.4)	6 (33.3)	0.694	32
Obesity, *n* (%)	8 (25.0)	2 (14.3)	6 (33.3)	0.412	32
Cardiovascular disease, *n* (%)	5 (15.6)	3 (21.4)	2 (11.1)	0.631	32
COPD, *n* (%)	9 (28.1)	6 (42.9)	3 (16.7)	0.132	32
Asthma, *n* (%)	2 (6.25)	1 (7.14)	1 (5.56)	1.000	32
Chronic kidney disease (60 mL/min/1.73 m^2^), *n* (%)	4 (12.5)	1 (7.14)	3 (16.7)	0.613	32
Chronic liver disease, *n* (%)	1 (3.12)	1 (7.14)	0 (0.0)	0.438	32
Autoimmune disease, *n* (%)	0 (0.0)	0 (0.0)	0 (0.0)	–	32
**ICU admission**					
Oxygen saturation (%), median [P25; P75]	90.0 [81.0; 92.0]	93.0 [87.0; 93.0]	89.5 [79.5; 90.2]	0.057	25
PaO_2_ (mmHg), median [P25; P75]	73.0 [55.0; 126]	122 [83.5; 140]	65.0 [48.8; 77.0]	0.012	30
PaCO_2_ (mmHg), median [P25; P75]	36.5 [30.5; 43.8]	39.5 [34.0; 47.8]	36.5 [28.2; 41.8]	0.280	30
Glucose (mg/dL), median [P25; P75]	130 [107; 152]	128 [110; 153]	131 [108; 150]	0.894	32
Creatinine (mg/dL), median [P25; P75]	0.88 [0.70; 1.17]	0.82 [0.70; 0.93]	0.98 [0.71; 1.35]	0.298	31
C-reactive protein (mg/L), median [P25; P75]	17.5 [8.64; 107]	8.64 [1.51; 35.7]	18.2 [10.8; 109]	0.204	24
LDH (U/L), mean (SD)	412 (183)	319 (172)	465 (173)	0.075	22
Leukocyte count (× 10^9^/L), mean (SD)	10.2 (5.64)	12.2 (7.10)	8.69 (3.69)	0.107	32
Neutrophil count (× 10^9^/L), median [P25; P75]	6.81 [5.00; 11.6]	13.6 [5.48; 15.3]	6.39 [4.92; 8.61]	0.075	30
Lymphocyte count (× 10^9^/L), median [P25; P75]	1.02 [0.49; 1.55]	1.44 [0.56; 1.87]	0.97 [0.48; 1.26]	0.241	30
Monocyte count (× 10^9^/L), median [P25; P75]	0.60 [0.43; 0.84]	0.65 [0.50; 0.97]	0.48 [0.39; 0.67]	0.191	29
Platelet count (× 10^9^/L), median [P25; P75]	240 [181; 281]	202 [146; 270]	256 [192; 298]	0.087	32
**ICU Stay**					
ICU stay (days), median [P25; P75]	30.0 [9.75; 58.0]	8.50 [6.25; 14.8]	54.0 [39.5; 79.0]	<0.001	32
ICU mortality, *n* (%)	9 (28.1)	6 (42.9)	3 (16.7)	0.132	32
Invasive mechanical ventilation, *n* (%)	31 (96.9)	13 (92.2)	18 (100)	0.438	32
Non-invasive mechanical ventilation, *n* (%)	24 (77.4)	7 (53.8)	17 (94.4)	0.012	31
Prone positioning, *n* (%)	16 (55.2)	1 (8.33)	15 (88.2)	<0.001	29
Antibiotics, *n* (%)	23 (71.9)	7 (50.0)	16 (88.9)	0.022	32
Hydroxychloroquine, *n* (%)	18 (56.2)	3 (21.4)	15 (83.3)	0.002	32
Tocilizumab, *n* (%)	8 (25.0)	2 (14.3)	6 (33.3)	0.412	32
Interferon beta, *n* (%)	1 (3.12)	0 (0.00)	1 (5.56)	1.000	32
Corticoids, *n* (%)	18 (56.2)	7 (50.0)	11 (61.1)	0.788	32
Remdesivir (negative), *n* (%)	32 (100)	14 (100)	18 (100)	–	32

*Continuous variables are expressed as the median [P25; P75] or mean (SD), and categorical variables are expressed as n (%). COPD, chronic obstructive pulmonary disease; ICU, intensive care unit; IMV, invasive mechanical ventilation; LDH, lactate dehydrogenase; PaCO_2_, carbon dioxide partial pressure; PaO_2_, oxygen partial pressure*.

**Figure 1:**
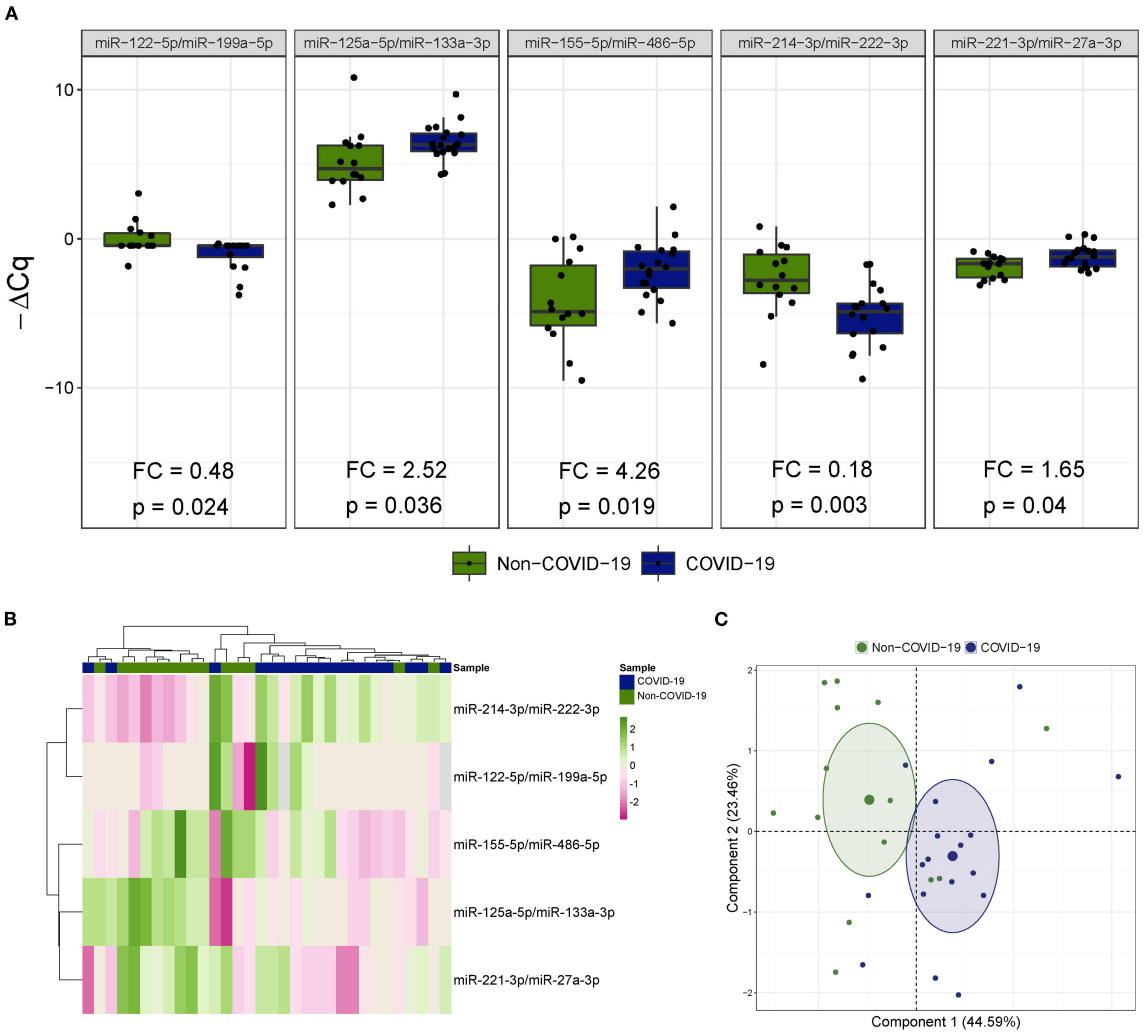
The microRNA ratio profile in bronchial aspirate samples from critically ill COVID-19 and non-COVID-19 patients. **(A)** Boxplot including those microRNA ratios that showed differences between critically ill COVID-19 and non-COVID-19 patients. Differences between groups were analyzed using linear models for arrays. *P*-values describe the significance level for each comparison; **(B)** Heatmap showing the unsupervised hierarchical clustering. Each column represents a patient (COVID-19 or non-COVID-19). Each row represents a microRNA. The patient clustering tree is shown on top. The microRNA ratio clustering tree is shown on the left. The color scale illustrates the relative expression level of microRNAs. Pink spectra represent increasing levels. Green spectra represent decreasing expression; **(C)** Principal component analysis. Each point represents a patient.

Five miRNA ratios were differentially detected (Figure 1A). Increased levels of miR-125a-5p/miR-133a-3p [fold change (FC) 2.52], miR-155-5p/miR-486-5p (FC 4.26) and miR-221-3p/miR-27a-3p (FC 1.65) were observed in the COVID-19 patients compared the patients not infected with SARS-CoV-2. In addition, miR-122-5p/miR-199a-5p (FC 0.48) and miR-214-3p/miR-222-3p (FC 0.18) were downregulated in patients with COVID-19-related critical illness. Unsupervised hierarchical clustering and PCA based on the expression profile of these ratios separated the COVID-19 patients from non-COVID-19 patients (Figures 1B,C). We further checked the influence of age and sex on the differences reported between the two study groups (Supplementary Table 3). No significant impact of either confounding factor was observed.

Gene functional enrichment analysis was performed to explore the molecular mechanisms related to the miRNA profile observed in BAS samples from COVID-19 patients (Supplementary Figures 1A,B and Supplementary Tables 4, 5). Thirty-seven KEGG pathways were enriched with the verified targets of the miRNA ratios, including “TGF-beta signaling pathway,” “AMPK signaling pathway,” “FoxO signaling pathway,” “HIF-1 signaling pathway,” “mTOR signaling pathway,” “p53 signaling pathway” and “ErbB signaling pathway.” One hundred and thirteen GO terms were identified. Processes included “viral process,” “viral life cycle,” “viral transcription,” “positive regulation of viral transcription,” “immune system process,” “innate immune response,” “leukocyte migration,” several signaling pathways linked to Toll-like receptors, “cell death” and several pathways related to apoptotic signaling, “blood coagulation,” “platelet activation” and “platelet degranulation.”

### The MicroRNA Profile of Bronchial Aspirates Is a Predictor of Fatal Outcome in Patients With COVID-19-Related Critical Illness

**Figure 2:**
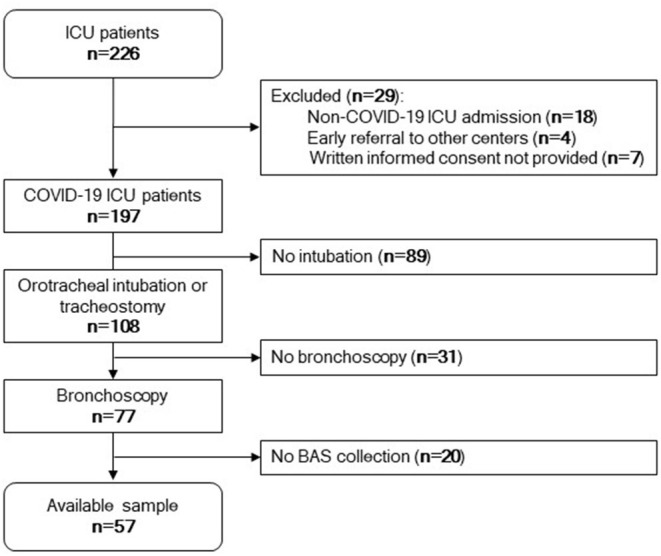
Study flowchart. Among 226 patients admitted to the ICU, 197 patients were positive for SARS-CoV-2. Eighteen were negative for SARS-CoV-2, four were referred to other centers early, and seven did not provide written informed consent. One hundred and eight patients were under invasive mechanical ventilation by tracheostomy or orotracheal intubation during the ICU stay. Among these, 77 patients were subjected to bronchoscopy. Fifty-seven samples were available for microRNA quantification.

Next, we sought to determine whether the miRNA signature of BAS samples can predict patient outcome. Figure 2 displays the study flowchart.

The patient demographics, clinical and pharmacological characteristics according to ICU survival are presented in Table 2. ICU mortality was 31.6% (*n* = 18/57). Non-survivors were typically older. At the time of ICU admission, non-survivors showed lower glucose concentrations. The days until IMV ventilation since the beginning of the symptoms and the hours under prone positioning were higher in those who eventually died. The causes of death were respiratory insufficiency (66.7%), multiorgan failure (27.8%) and cardiovascular accidents (5.5%). The comparison between those patients with BAS samples and all patients under IMV or patients under IMV without BAS samples is displayed in Supplementary Tables 6, 7. Microbiological and fungal examinations showed no differences between the study groups (Supplementary Table 8).

**Table 2:** Characteristics of patients with COVID-19-related critical illness who survived the ICU stay or not (Study Population 2).

	**ALL**	**Survivor**	**Non-survivor**	***p*-value**	**Available**
	***n* = 57**	***n* = 39**	***n* = 18**		**data**
**Sociodemographic characteristics**					
Age (years), median [P25; P75]	63.0 [59.0; 70.0]	62.0 [56.0; 69.0]	65.5 [63.0; 71.0]	0.089	57
Male, *n* (%)	46 (80.7)	32 (82.1)	14 (77.8)	0.728	57
Smoking history, *n* (%)				0.913	51
Former	24 (47.1)	17 (48.6)	7 (43.8)		
Non-smoker	22 (43.1)	15 (42.9)	7 (43.8)		
Current	5 (9.80)	3 (8.57)	2 (12.5)		
Alcoholism, *n* (%)	4 (7.41)	3 (8.11)	1 (5.88)	0.809	54
**Comorbidities**					
Hypertension, *n* (%)	34 (59.6)	24 (61.5)	10 (55.6)	0.891	57
Type II Diabetes Mellitus, *n* (%)	22 (38.6)	18 (46.2)	4 (22.2)	0.152	57
Obesity, *n* (%)	33 (57.9)	22 (56.4)	11 (61.1)	0.964	57
Cardiovascular disease, *n* (%)	10 (17.5)	7 (17.9)	3 (16.7)	1.000	57
COPD, *n* (%)	5 (8.77)	2 (5.13)	3 (16.7)	0.312	57
Asthma, *n* (%)	3 (5.26)	3 (7.69)	0 (0.00)	0.544	57
Chronic kidney disease (60 mL/min/1.73 m^2^), *n* (%)	2 (3.51)	2 (5.13)	0 (0.00)	1.000	57
Chronic liver disease, *n* (%)	3 (5.26)	2 (5.13)	1 (5.56)	1.000	57
Autoimmune disease, *n* (%)	1 (1.75)	1 (2.56)	0 (0.00)	1.000	57
**ICU admission**					
Time since first symptoms to hospital admission (days), median [P25; P75]	7.00 [4.00; 8.00]	5.00 [4.00; 8.00]	7.00 [5.25; 11.0]	0.094	57
Time since first symptoms to ICU admission (days), median [P25; P75]	8.00 [6.00; 10.0]	7.00 [5.00; 9.50]	10.0 [7.00; 12.8]	0.062	57
Time since hospital admission to ICU admission (days), median [P25; P75]	1.00 [0.00; 3.00]	0.00 [0.00; 3.00]	1.00 [0.00; 2.75]	0.862	57
Oxygen saturation (%), median [P25; P75]	94.0 [89.8; 95.9]	93.8 [88.0; 95.5]	94.9 [91.2; 96.8]	0.160	56
FiO_2_ (%), median [P25; P75]	70.0 [50.0; 90.0]	60.0 [50.0; 90.0]	70.0 [60.0; 89.5]	0.278	56
PaO_2_ (mmHg), median [P25; P75]	69.0 [52.5; 83.8]	63.0 [49.0; 72.8]	79.0 [67.2; 86.2]	0.087	42
PaCO_2_ (mmHg), median [P25; P75]	37.0 [32.0; 41.0]	38.0 [33.0; 44.8]	32.5 [29.8; 36.2]	0.100	42
PaO_2_/FiO_2_, median [P25; P75]	108 [78.3; 156]	94.0 [78.3; 151]	116 [81.6; 155]	0.738	42
SaO_2_/FiO_2_, median [P25; P75]	139 [105; 172]	147 [106; 189]	132 [103; 156]	0.301	56
Glucose (mg/dL), median [P25; P75]	160 [121; 228]	190 [124; 246]	126 [114; 166]	0.025	57
Creatinine (mg/dL), median [P25; P75]	0.84 [0.69; 1.13]	0.84 [0.70; 1.03]	0.84 [0.68; 1.13]	0.952	57
C-reactive protein (mg/L), median [P25; P75]	152 [66.0; 197]	152 [74.3; 194]	152 [56.0; 215]	0.888	56
D-dimer (ng/mL), median [P25; P75]	393 [285; 728]	394 [287; 1081]	367 [285; 437]	0.265	48
Leukocyte count (× 10^9^/L), median [P25; P75]	7.67 [6.37; 10.2]	7.33 [6.28; 8.54]	8.72 [6.51; 11.9]	0.127	57
Neutrophil count (× 10^9^/L), median [P25; P75]	6.43 [5.00; 8.45]	6.30 [5.14; 7.53]	7.10 [5.06; 9.18]	0.363	57
Lymphocyte count (× 10^9^/L), median [P25; P75]	0.65 [0.51; 1.02]	0.64 [0.52; 1.00]	0.70 [0.51; 1.10]	0.600	57
Monocyte count (× 10^9^/L), median [P25; P75]	0.32 [0.20; 0.47]	0.29 [0.19; 0.38]	0.41 [0.21; 0.51]	0.135	57
Platelet count (× 10^9^/L), mean (SD)	216 (67.7)	217 (55.0)	214 (91.3)	0.886	57
AST (U/L), median [P25; P75]	60.0 [38.0; 89.0]	60.0 [38.0; 85.0]	60.0 [39.8; 92.2]	0.879	39
ALT (U/L), median [P25; P75]	37.0 [27.0; 59.0]	44.0 [26.0; 61.5]	36.0 [28.8; 47.0]	0.648	39
Urea (mg/dL), median [P25; P75]	51.0 [40.0; 62.0]	50.0 [33.0; 61.5]	59.5 [46.8; 66.8]	0.116	57
APACHE-II score, median [P25; P75]	16.0 [13.0; 20.0]	15.5 [12.2; 20.0]	16.5 [15.0; 19.8]	0.342	56
**ICU Stay**					
ICU stay (days), median [P25; P75]	32.0 [16.0; 48.0]	32.0 [16.0; 51.0]	31.5 [21.5; 46.8]	0.857	57
Invasive mechanical ventilation duration (days), median [P25; P75]	27.0 [14.0; 40.0]	23.0 [12.0; 41.0]	30.0 [23.8; 39.8]	0.327	57
Prone positioning, *n* (%)	50 (87.7)	33 (84.6)	17 (94.4)	0.413	57
Prone positioning duration (hours), median [P25; P75]	64.0 [33.5; 136]	44.0 [27.8; 102]	135 [94.5; 232]	0.005	47
Antibiotics, *n* (%)	54 (94.7)	38 (97.4)	16 (88.9)	0.232	57
Hydroxychloroquine, *n* (%)	8 (14.0)	5 (12.8)	3 (16.7)	0.698	57
Tocilizumab, *n* (%)	44 (77.2)	32 (82.1)	12 (66.7)	0.308	57
Corticoids, *n* (%)	55 (98.2)	38 (100)	17 (94.4)	0.321	56
**Initiation of invasive mechanical ventilation**					
Time since first symptoms to IMV (days), median [P25; P75]	10.0 [7.00; 13.0]	8.00 [6.50; 11.0]	13.0 [10.0; 15.8]	0.006	57
Time since hospital admission to IMV (days), median [P25; P75]	2.00 [0.00; 6.00]	2.00 [0.00; 5.50]	3.00 [1.00; 7.00]	0.222	57
Time since ICU admission to IMV (days), median [P25; P75]	1.00 [0.00; 3.00]	1.00 [0.00; 2.00]	1.50 [0.00; 4.00]	0.122	57
Oxygen saturation (%), median [P25; P75]	95.1 [92.0; 98.0]	95.0 [90.8; 97.0]	97.0 [94.7; 99.0]	0.144	55
FiO_2_ (%), median [P25; P75]	70.0 [50.0; 90.0]	60.0 [50.0; 83.8]	70.0 [50.0; 100]	0.420	55
PaO_2_ (mmHg), median [P25; P75]	95.0 [70.0; 115]	90.0 [63.0; 110]	103 [82.0; 116]	0.220	40
PaCO_2_ (mmHg), mean (SD)	45.7 (12.1)	46.1 (12.1)	44.7 (12.5)	0.730	40
PaO_2_/FiO_2_, mean (SD)	204 (92.3)	221 (97.0)	178 (84.2)	0.310	20
SaO_2_/FiO_2_, mean (SD)	173 (51.5)	182 (51.0)	160 (52.9)	0.377	20
Glucose (mg/dL), median [P25; P75]	146 [114; 211]	160 [122; 233]	126 [110; 164]	0.096	56
Creatinine (mg/dL), median [P25; P75]	0.78 [0.65; 1.00]	0.78 [0.67; 0.99]	0.77 [0.65; 1.07]	0.880	56
C-reactive protein (mg/L), median [P25; P75]	136 [19.9; 210]	147 [24.1; 197]	67.7 [17.7; 234]	0.985	55
D-dimer (ng/mL), median [P25; P75]	754 [296; 2172]	858 [298; 2153]	619 [364; 2010]	0.883	41
Leukocyte count (x10^9^/L), median [P25; P75]	8.19 [6.29; 11.6]	7.67 [6.27; 10.8]	9.83 [7.84; 13.1]	0.119	56
Neutrophil count (x10^9^/L), median [P25; P75]	7.30 [5.34; 9.93]	6.41 [5.32; 9.38]	8.07 [5.86; 9.93]	0.301	56
Lymphocyte count (× 10^9^/L), median [P25; P75]	0.70 [0.58; 0.99]	0.68 [0.52; 0.95]	0.78 [0.64; 1.09]	0.203	56
Monocyte count (× 10^9^/L), median [P25; P75]	0.31 [0.18; 0.48]	0.29 [0.18; 0.38]	0.38 [0.18; 0.54]	0.190	56
Platelet count (× 10^9^/L), mean (SD)	230 (67.2)	231 (57.0)	227 (88.4)	0.874	56
AST (U/L), median [P25; P75]	60.0 [37.8; 87.0]	53.5 [36.8; 79.0]	77.0 [43.8; 91.2]	0.240	36
ALT (U/L), median [P25; P75]	38.5 [27.5; 55.5]	32.5 [23.8; 55.5]	43.5 [36.5; 57.0]	0.127	36
Urea (mg/dL), median [P25; P75]	49.5 [36.0; 64.5]	50.0 [34.5; 63.0]	49.0 [38.0; 66.0]	0.656	56

*Continuous variables are expressed as the median [P25; P75] or mean (SD) and categorical variables are expressed as n (%). ALT, alanine aminotransferase; AST, aspartate aminotransferase; COPD, chronic obstructive pulmonary disease; FiO_2_, fraction of inspired oxygen; ICU, intensive care unit; IMV, invasive mechanical ventilation; LDH, lactate dehydrogenase; PaCO_2_, carbon dioxide partial pressure; PaO_2_, oxygen partial pressure; SaO_2_, oxygen saturation*.

**Figure 3:**
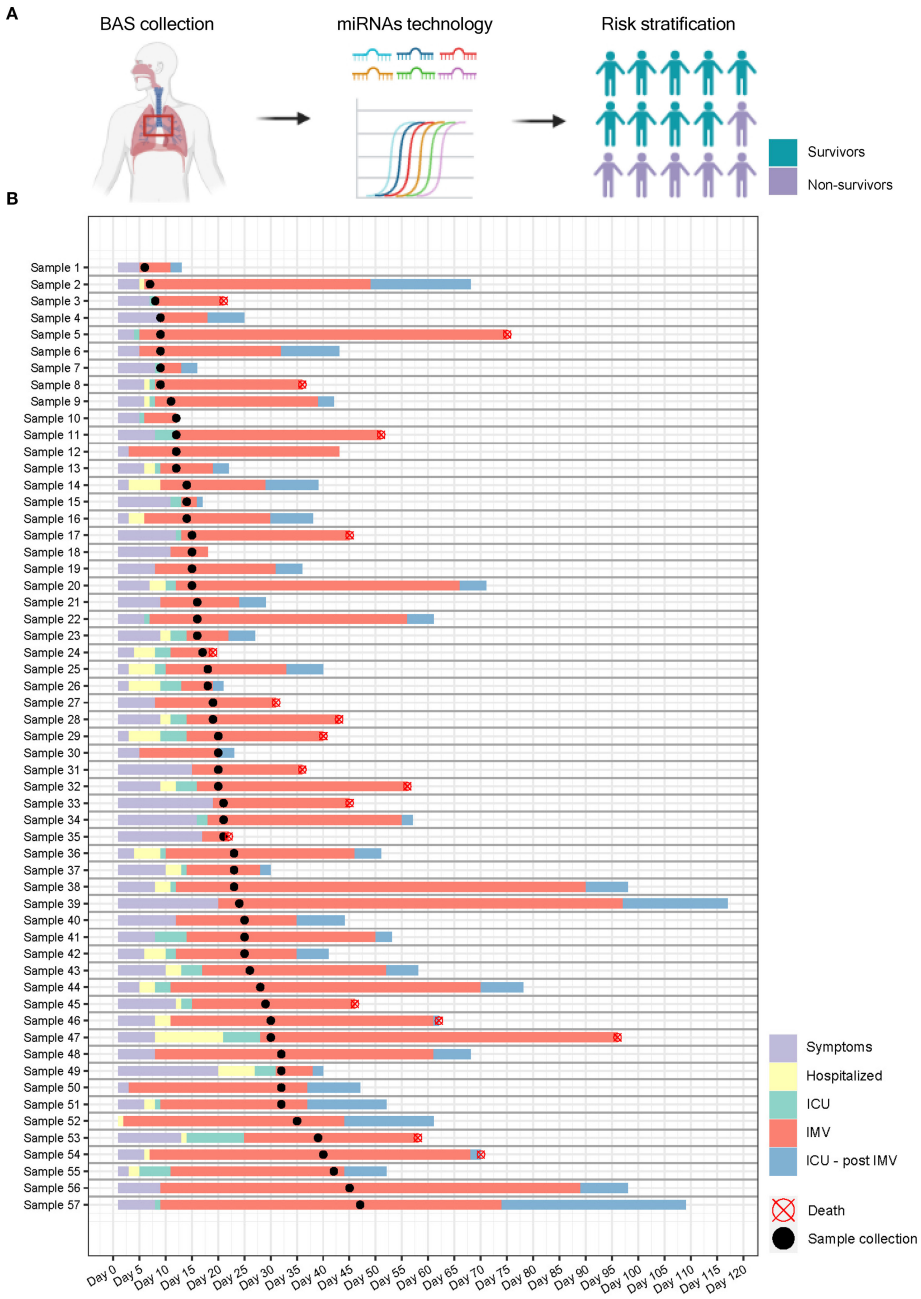
Study design and sample collection. **(A)** microRNA ratios were quantified in bronchial aspirate samples from critically ill COVID-19 patients using RT-qPCR. The final aim is to identify microRNA signatures as prognostic biomarkers of fatal outcome in COVID-19 and to define molecular pathways involved in the development of adverse events; **(B)** Timeline. Each row represents a patient. Information regarding symptoms, hospitalization, ICU stay, invasive mechanical ventilation and postinvasive mechanical ventilation periods is displayed.

**Figure 4:**
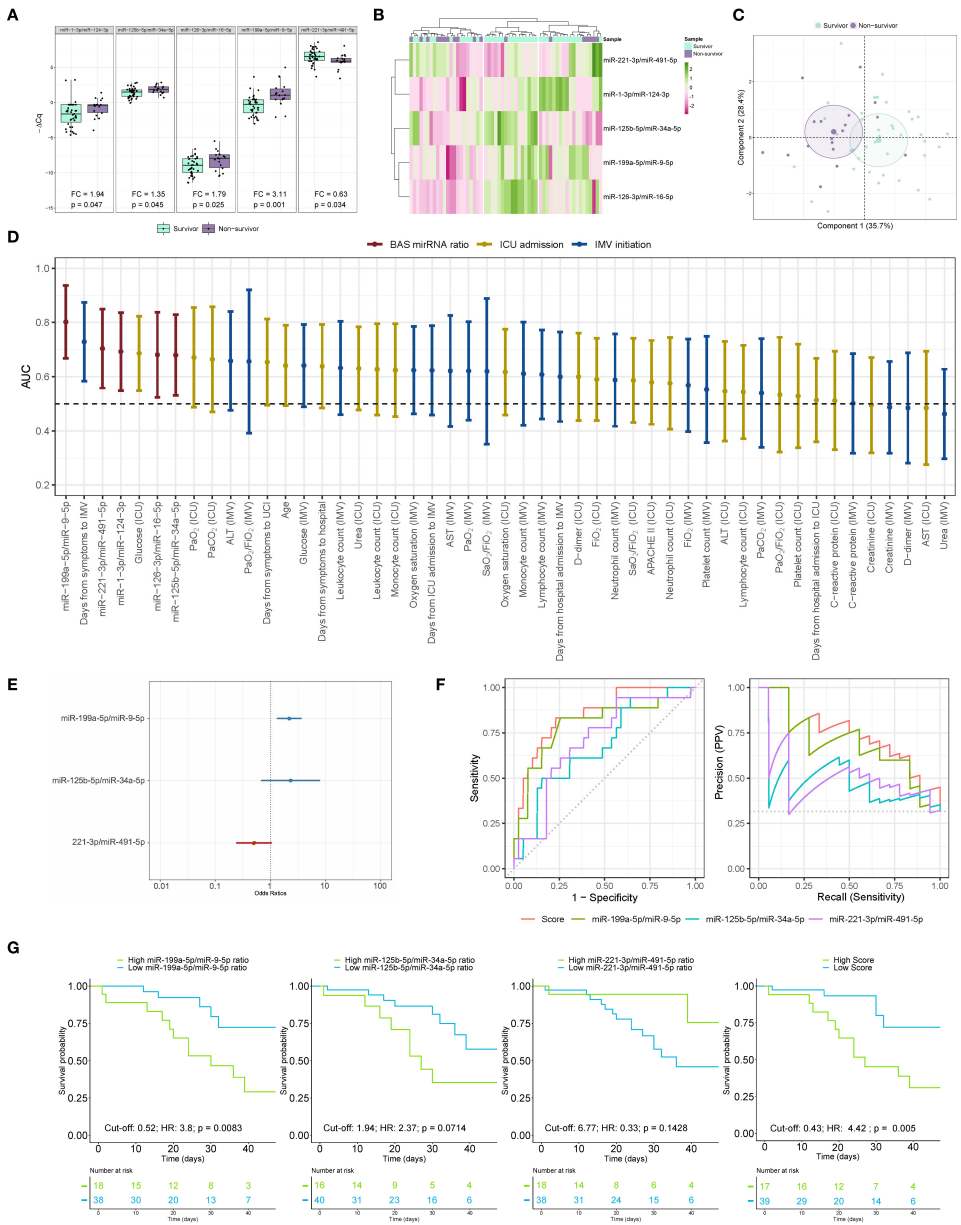
The microRNA ratio profile in bronchial aspirate samples from COVID-19 survivors and nonsurvivors to ICU stay. **(A)** Boxplot including the microRNA ratios that showed differences between survivors and non-survivors. Differences between groups were analyzed using linear models for arrays. P-values describe the significance level for each comparison; **(B)** Heatmap showing the unsupervised hierarchical clustering. Each column represents a patient (survivor or non-survivor). Each row represents a microRNA. The patient clustering tree is shown on top. The microRNA ratio clustering tree is shown on the left. The color scale illustrates the relative expression level of microRNAs. Pink spectra represent increasing levels. Green spectra represent decreasing expression; **(C)** Principal component analysis. Each point represents a patient. **(D)** Discrimination value for microRNA ratios and clinical predictors. The data are presented as the AUC and 95% CI; **(E)** The microRNA ratio-based prediction model (microRNA ratio score); **(F)** ROC curves and precision-recall curves for individual microRNA ratios and miRNA ratio score; **(G)** Kaplan-Meier estimations for the individual miRNA ratios and microRNA ratio score. Cut-off, hazard ratios and *p*-values are displayed. ALT, alanine aminotransferase; AST, aspartate aminotransferase; FiO_2_, fraction of inspired oxygen; ICU, intensive care unit; IMV, invasive mechanical ventilation; PaCO_2_, carbon dioxide partial pressure; PaO_2_, oxygen partial pressure; SaO_2_, oxygen saturation.

BAS samples were collected for miRNA quantification from 57 patients within a median [IQR] of 6 [10] days from the beginning of IMV (Figure 3). As shown in Figure 4A, patients who died had a significant upregulation in the ratios of miR-1-3p/miR-124-3p (FC 1.94), miR-125b-5p/miR-34a-5p (FC 1.35), miR-126-3p/miR-16-5p (FC 1.79) and miR-199a-5p/miR-9-5p (FC 3.11). Conversely, the levels of miR-221-3p/miR-491-5p (FC 0.63) were reduced in non-survivors. Unsupervised hierarchical clustering and PCA based on the expression profile of these five miRNA ratios separated the survivors from non-survivors (Figures 4B,C). We analyzed the influence of age and sex on the miRNA profile. No substantial impact of either confounding factor on the differences between study groups was observed (Supplementary Table 9).

Then, ROC curves and AUCs were used to assess the discriminative accuracy of the BAS ratios (Figure 4D). The AUC for discriminating survivors vs. non-survivors was optimal for the miR-199a-5p/miR-9-5p ratio (AUC 0.80). The miR-1-3p/miR-124-3p, miR-125b-5p/miR-34a-5p, miR-126-3p/miR-16-5p and miR-221-3p/miR-491-5p ratios showed AUCs from 0.68 to 0.70. The AUC for miR-199a-5p/miR-9-5p was greater than that observed for the best clinical predictor: the time from first symptoms to IMV initiation (AUC 0.73). The five ratios were among the top seven predictors of mortality during the ICU stay.

A predictor selection procedure based on random forest was used to select the optimal combination of ratios for the prediction of mortality during the ICU stay. The multivariable analysis selected a 3-miRNA ratio prediction model (miRNA ratio score) composed of miR-125b-5p/miR-34a-5p, miR-199a-5p/miR-9-5p and miR-221-3p/miR-491-5p ratios (Figure 4E). The discriminatory capacity of the score composed of the three ratios was higher than that observed for individual miRNA ratios or clinical predictors (AUC 0.85) (Figure 4D). The performance of the miRNA ratio score was the highest in the precision-recall curves (Figure 4F). The Kaplan-Meier analysis showed that patients with high levels in the score based on the 3-miRNA ratio model and the individual miR-199a-5p/miR-9-5p ratio were at significantly higher risk of in-ICU death (HR 4.42 and HR 3.8, respectively) (Figure 4G). An inverse correlation between the ratio miR-1-3p/miR-124-3p and the number of days under IMV was observed (Supplementary Figure 2).

*In silico* analysis was performed to identify the molecular pathways and biological processes most closely linked to the miRNA profile from non-survivors (Supplementary Figures 3A,B and Supplementary Tables 10, 11). Twenty-five KEGG pathways and 122 GO terms were enriched with the verified targets of miRNAs that comprised the ratios. Pathways and GO terms related to COVID-19 pathophysiology were identified, including the “TGF-beta signaling pathway,” “viral process,” “viral life cycle,” “viral transcription,” “blood coagulation,” “platelet activation,” “platelet degranulation,” “immune system process,” “positive regulation of type I interferon production,” “innate immune response” and “leukocyte migration,” “cell death” and several signaling pathways linked to Toll-like receptors and apoptosis.

## Discussion

Significant advancements in the outcomes and treatment of patients with COVID-19 have been made since the beginning of the pandemic. Nevertheless, there is considerable room to improve the risk stratification and therapeutic management of patients with COVID-19-related critical illness (4). As such, we analyzed the host miRNA profile of BAS samples from COVID-19 and non-COVID-19 patients admitted to the ICU. We report that (i) the miRNA signature of critically ill COVID-19 patients is essentially different from that observed in non-COVID-19 patients; (ii) non-survivors of an ICU stay show a characteristic miRNA profile when compared to that of survivors; (iii) miRNA profiling in respiratory specimens allows risk stratification in COVID-19 patients under IMV; and iv) the miRNA profile provides information about the factors that mediate the disease and fatal outcomes among critically ill COVID-19 patients.

### Clinical Implications for the Management of Critically Ill Patients With COVID-19 Assisted by Invasive Mechanical Ventilation

Data regarding the host miRNA profile within the lung in COVID-19 patients are limited. Aberrant individual miRNA or miRNA profiles in the lung compartment have been observed in respiratory viral infections, such as influenza A virus (27). Since the beginning of the pandemic, most studies have explored alterations in the miRNA profile of matrices such as whole blood, plasma and serum (28–31). However, the identification of biomarkers for lung disease using blood samples is controversial, at least in pneumonia-related ARDS. Lung compartmentalization exists for specific pathological mechanisms, e.g., inflammation (32). From a practical standpoint, miRNA assessment in BAS emerges as an interesting tool to define phenotypes among ICU patients under IMV. Of note, BAS may reflect *in situ* host response against SARS-CoV-2 infection in the lung, is relatively easy to collect and can replace other procedures that are not well-tolerated by the patient, such as BALF collection (33).

Our results demonstrated for the first time the biomarker potential of miRNAs in BAS samples. To our knowledge, this is also the first study that reports host miRNA profiles in respiratory specimens as predictors of clinical outcomes in COVID-19-related critical illness. Critically ill patients with COVID-19 under IMV can be classified by multi miRNA signatures quantified in the respiratory tract. In particular, five miRNA ratios segregated between ICU survivors and non-survivors. Simultaneous assessment of miRNA profiles or miRNA ratios may hold promise to provide more comprehensive information about the clinical evolution of the patient. This hypothesis, which seems to be true for circulating miRNAs (34), can also be translated to respiratory specimens such as BAS. Using multivariable analysis for multimarker analysis, we constructed a miRNA ratio-based prediction model (miRNA ratio score) to optimize the best combination of ratios for risk prediction. The score based upon miRNA expression ratios (miR-125b-5p/miR-34a-5p, miR-199a-5p/miR-9-5p, and miR-221-3p/miR-491-5p) and the individual miR-199a-5p/miR-9-5p ratio showed excellent discrimination value; AUC 0.85 and 0.80, respectively. The survival analysis confirmed the great potential of the miRNA ratio score and the miR-199a-5p/miR-9-5p ratio for identifying patients at very high risk of fatal outcomes following IMV initiation.

To date, the prognosis of critically ill patients is mostly based on clinical characterization. Consequently, the estimated ratios were also compared with sociodemographic variables, clinical parameters or laboratory parameters, including a comprehensive set of indicators collected at the time of ICU admission or initiation of IMV. Some of these parameters, including the PaO_2_:FiO_2_ ratio, leukocyte counts and D-dimer, C-reactive protein (CRP) and creatinine concentrations, among others, have been previously reported as mortality predictors in ventilated and/or ICU COVID-19 patients (2, 35–37). The miRNA ratio signature and the miR-199a-5p/miR-9-5p ratio outperformed the best clinical predictor for ICU mortality (days from first symptoms to IMV initiation, AUC 0.73).

Based on these findings, miRNA quantification in BAS samples may support patient stratification and constitute an objective tool for individualizing and guiding clinical care during the ICU stay, e.g., treatment replacement and the use of novel medications in addition to conventional medical treatment or more intensive monitoring and allocation of hospital resources.

### Molecular Mechanisms Associated With COVID-19 and Fatal Courses of the Disease

Previous investigations have proposed crucial roles of ncRNAs in the pathophysiology of COVID-19 (29). Since these RNAs, such as miRNAs, can be measured in respiratory secretions, ncRNAs constitute an interesting tool to molecularly phenotype patients. The current results may improve the limited understanding of SARS-CoV-2 pathogenesis and the host biological response. This information is particularly relevant due to the atypical features linked to critically ill COVID-19 patients. Notably, miRNA targeting has been previously reported as an interesting approach for the treatment of viral infections (38) and chronic disease (39).

We report that SARS-CoV-2 infection induces a unique miRNA profile in BAS samples from COVID-19 patients. Five miRNA ratios distinguished between laboratory-confirmed infected and non-infected patients. Functional enrichment analyses using experimentally validated miRNA:gene interactions suggested the implication of these transcripts in different pathophysiological axes of the disease and are in line with previous findings that demonstrated a characteristic immunoinflammatory profile of COVID-19 patients (40). A number of pathways and GO terms were associated with mechanisms such as immune cell differentiation and activation, cytokine and chemokine synthesis and the regulation of the inflammatory process. Several GO terms were linked to the coagulation process, a hallmark of COVID-19. Notably, the ratio containing miR-155-5p, proposed as a key regulator of T-cell maturation and the innate immune response (41), showed significant differences between the study groups. Some of these miRNAs are also known as mediators of viral infections. Hepatitis C virus uses miR-122 to facilitate its replication (38). In addition, miR-155 expression is required for the growth of Epstein-Barr virus-infected B cells (42). Interestingly, infection by SARS-CoV in bronchoalveolar stem cells causes miR-214 overexpression, presented in the ratio that showed higher differences, which has been described as a mechanism for evading immune elimination until successful transmission takes place (43).

Additionally, miRNA ratios may be informative about the driving factors and triggers of fatal COVID-19 forms. miR-199-5p has been described as a miRNA with pro-viral functions. For instance, it enhances hepatitis C virus replication, and its downregulation suppresses virus replication in an *in vitro* knockdown model (44). Furthermore, miR-199-5p, together with miR-214-3p, has also been proposed as a major regulator of lung fibrosis. Independent investigations using *in vitro* and *in vivo* models reported this miRNA as a key effector of TGF-β-induced lung myofibroblast activation (45, 46). Pulmonary miR-199-5p expression is increased in patients with idiopathic forms of fibrotic lung disease (46), and its extracellular levels are elevated in bodily fluids (e.g., sputum) in respiratory conditions (47). The possible role of miR-9-5p in SARS-CoV-2 infectivity should not be disregarded. Angiotensin-converting enzyme 2 (ACE2) mRNA presents high-probability miRNA binding sites for this transcript in the 3′-UTR (48). Supporting the participation of miR-9-5p in the regulation of coronavirus infections, the human coronavirus OC43 nucleocapsid protein binds miR-9, a negative regulator of the multifunctional transcription factor NF-κB, which ultimately impacts the regulation of the inflammatory response to viral infection (49). In general, there is a preponderance of miRNAs related to inflammatory mechanisms. miR-16-5p, miR-124-3p, miR-125b-5p, miR-126-3p, and miR-221-3p are able to target molecules belonging to pathways with a key role in inflammatory responses, including receptors (TLRs), signaling molecules (MYD88), transcription factors (NF-κB) and cytokines/chemokines (TNF, IL-6 and IL-8) (50–53). Some miRNAs, such as miR-126-5p and miR-221-3p, may be associated with the severe endothelial injury and coagulopathic features observed in lung samples from fatal cases (54). Both miRNAs, in addition to miR-16-5p, may also be linked to the vascular angiogenesis previously described in the lungs of patients who have succumbed to the disease (55, 56). In the context of coagulation, recent findings demonstrated that miR-34a-5p is downregulated in postmortem lung biopsies of COVID-19 patients with severe respiratory injuries and thrombotic events (57). The miRNA target sites (MTSs) within SARS-CoV-2 have been identified for this miRNA (58). The same authors suggested that the virus genome may modulate the levels of miR-34a-5p, acting as a miRNA sponge to regulate unfolded protein response (UPR)-related apoptosis. The KEGG and GO analyses support the participation of these host miRNAs in the pathogenesis of the disease.

Overall, the findings add additional information to genomic, proteomic, metabolic and cellular data to identify new drug targets or therapeutic agents (59). This study provides useful hypothesis-generating data and constitutes a basis for future investigations in critically ill patients. The miRNAs described in the current study deserve additional analyses as therapeutic targets/agents. The role as mediators or consequence of the disease and the pathophysiological importance in fatal outcomes should be explored in detail using alternative approaches, including mechanistic *in vitro* and *in vivo* studies.

### Strengths and Limitations of the Study

The strengths of the study include the rigorous quality control of clinical and miRNA data, the exploration of a real-world setting that included consecutive patients who underwent bronchoscopy and the evaluation of the miRNA ratios in conjunction with comprehensive clinical information. Nevertheless, the conclusions should be interpreted in the context of several limitations. First, mortality was evaluated in a single-center study that included a specific subpopulation of ICU patients, i.e., patients assisted by IMV and subjected to bronchoscopy. Although the highest mortality rates during the pandemic have been concentrated in the group of patients who require IMV secondary to ARDS, the predictive power of the miRNA signature remains to be validated in alternative patient subgroups. Second, the impact of the disease on the miRNA profile and the potential of these small transcripts as prognostic biomarkers need to be confirmed in larger populations. Third, the non-COVID-19 group was composed of patients presenting heterogeneous causes of ICU admission. The main causes of ICU admission among non-COVID-19 patients were pneumonia (35.7%) and brain edema (14.3%) (Supplementary Table 2). The inclusion of a group of patients with viral pneumonia constitutes an interesting approach to characterize with more detail the molecular impact of SARS-CoV-2 infection. Forth, we could not exclude the impact of complications and treatments during ICU stay that were not recorded. Fifth, the source for the miRNA profile in BAS is not fully defined and could reflect the intracellular profile of or release from immune, pulmonary, epithelial or vascular cells. It is worth noting that the contribution of tissue damage to the BAS miRNA profile should not be disregarded. MiRNAs are passively released to the extracellular space during cell death, with cardiomyocyte-specific miRNAs; i.e., miR-208a-3p and miR-208b-3p, as familiar examples (60). Additional approaches are fundamental to infer any causal association. Sixth, miRNA profiling in BAS is challenging because of the extraordinary complexity and broad dynamic range of miRNA concentrations. BAS shows a high sample-to-sample variation, which is due to the collection of the sample during bronchoaspiration (e.g., bronchial washing with saline solution) that cannot be accounted for using standard normalization methods. The comparison of individual miRNAs between BAS samples is not possible. To overcome this limitation, we analyzed miRNA ratios, which precludes the need for normalization factors instead of individual miRNAs.

## Conclusions

COVID-19 induces a specific host miRNA profile in BAS samples from critically ill patients. Furthermore, specific miRNA ratios in respiratory secretions, particularly BAS, show individual and collective potential for risk-based patient stratification following IMV initiation in COVID-19-related critical illness. The biological role of the miRNA profiles in BAS samples allows a better understanding of the different pathological axes of the disease. The role of miRNAs as mediators and biomarkers should be further explored.

## Data Availability Statement

The original contributions presented in the study are included in the article/Supplementary Files, further inquiries can be directed to the corresponding author/s.

## Ethics Statement

Samples were processed following standard operating procedures with the appropriate approval of the Ethics and Scientific Committees (s007-BBCOV) and with the collaboration of the healthcare services of the Hospital Universitario Son Espases and Hospital Son Llatzer (Palma, Spain).

## Author Contributions

JC, JFB-M, AC, LF-B, RF, DG-G, RM, AM, OP, JR, AT, FB, and DdG-C contributed to the study concept and design. MM, JG, CG-P, AM-M, FR-J, MG-H, GT, JV, SG, and RC contributed to the data acquisition. MM, IB, JG, GT, JC, JFB-M, AC, LF-B, RF, DG-G, RM, AM, OP, JR, AT, FB, and DdG-C contributed to the data analysis and interpretation. DdG-C is the guarantor of the paper, had full access to all of the data in the study, and takes responsibility for the integrity of the data and the accuracy of the data analysis. All authors contributed to the manuscript draft, critically revised the manuscript for important intellectual content, and approved the final version.

## Funding

This work was supported by Instituto de Salud Carlos III (COV20/00110), co-funded by European Regional Development Fund (ERDF)/A way to make Europe. CIBERES is an initiative of the Instituto de Salud Carlos III. Supported by: Programa de donaciones estar preparados; UNESPA (Madrid, Spain). DdG-C (Miguel Servet 2020: CP20/00041) and MM (PFIS: FI21/00187) have received financial support from the Instituto de Salud Carlos III, co-funded by the European Social Fund (ESF)/Investing in your future. MM is the recipient of a predoctoral fellowship (PERIS, PIF-Salut, SLT017/20/000142) from the Department de Salut (Generalitat de Catalunya). MG-H is the recipient of a predoctoral fellowship from University of Lleida.

## Conflict of Interest

DdG-C holds patents on microRNAs as biomarkers. The remaining authors declare that the research was conducted in the absence of any commercial or financial relationships that could be construed as a potential conflict of interest.

## Publisher's Note

All claims expressed in this article are solely those of the authors and do not necessarily represent those of their affiliated organizations, or those of the publisher, the editors and the reviewers. Any product that may be evaluated in this article, or claim that may be made by its manufacturer, is not guaranteed or endorsed by the publisher.
