# Supplementary material for: Bronchial Aspirate-Based Profiling Identifies MicroRNA Signatures Associated With COVID-19 and Fatal Disease in Critically Ill Patients
Source: Front Med (Lausanne). 2022 Feb 3;8:756517. doi: 10.3389/fmed.2021.756517 (PMC8850692; doi:10.3389/fmed.2021.756517)
Supplement: Supplementary file 1 [file Data_Sheet_1.docx]

**SUPPLEMENTARY MATERIAL**

**SUPPLEMENTAL TABLES**

| **Table S1.** microRNA panel. | | | |
| --- | --- | --- | --- |
|  |  |  |  |
| **microRNA ID** | **miRBase Accession Number** | **Target sequence** | **MiRCury Assay** |
|  |  |  |  |
| hsa-miR-1-3p | MIMAT0000416 | UGGAAUGUAAAGAAGUAUGUAU | YP00204344 |
| hsa-miR-9-5p | MIMAT0000441 | UCUUUGGUUAUCUAGCUGUAUGA | YP00204513 |
| hsa-miR-16-5p | MIMAT0000069 | UAGCAGCACGUAAAUAUUGGCG | YP00205702 |
| hsa-miR-17-5p | MIMAT0000070 | CAAAGUGCUUACAGUGCAGGUAG | YP02119304 |
| hsa-miR-21-5p | MIMAT0000076 | UAGCUUAUCAGACUGAUGUUGA | YP00204230 |
| hsa-miR-24-3p | MIMAT0000080 | UGGCUCAGUUCAGCAGGAACAG | YP00204260 |
| hsa-miR-27a-3p | MIMAT0000084 | UUCACAGUGGCUAAGUUCCGC | YP00206038 |
| hsa-miR-27b-3p | MIMAT0000419 | UUCACAGUGGCUAAGUUCUGC | YP00205915 |
| hsa-miR-34a-5p | MIMAT0000255 | UGGCAGUGUCUUAGCUGGUUGU | YP00204486 |
| hsa-miR-34b-5p | MIMAT0000685 | UAGGCAGUGUCAUUAGCUGAUUG | YP00204424 |
| hsa-miR-34c-5p | MIMAT0000686 | AGGCAGUGUAGUUAGCUGAUUGC | YP00205659 |
| hsa-miR-92a-3p | MIMAT0000092 | UAUUGCACUUGUCCCGGCCUGU | YP00204258 |
| hsa-miR-93-5p | MIMAT0000093 | CAAAGUGCUGUUCGUGCAGGUAG | YP00204715 |
| hsa-miR-98-5p | MIMAT0000096 | UGAGGUAGUAAGUUGUAUUGUU | YP00204640 |
| hsa-miR-122-5p | MIMAT0000421 | UGGAGUGUGACAAUGGUGUUUG | YP00205664 |
| hsa-miR-124-3p | MIMAT0000422 | UAAGGCACGCGGUGAAUGCC | YP00206026 |
| hsa-miR-125a-5p | MIMAT0000443 | UCCCUGAGACCCUUUAACCUGUGA | YP00204339 |
| hsa-miR-125b-5p | MIMAT0000423 | UCCCUGAGACCCUAACUUGUGA | YP00205713 |
| hsa-miR-126-3p | MIMAT0000445 | UCGUACCGUGAGUAAUAAUGCG | YP00204227 |
| hsa-miR-132-3p | MIMAT0000426 | UAACAGUCUACAGCCAUGGUCG | YP00206035 |
| hsa-miR-133a-3p | MIMAT0000427 | UUUGGUCCCCUUCAACCAGCUG | YP00204788 |
| hsa-miR-146a-5p | MIMAT0000449 | UGAGAACUGAAUUCCAUGGGUU | YP00204688 |
| hsa-miR-148a-3p | MIMAT0000243 | UCAGUGCACUACAGAACUUUGU | YP00205867 |
| hsa-miR-150-5p | MIMAT0000451 | UCUCCCAACCCUUGUACCAGUG | YP00204660 |
| hsa-miR-155-5p | MIMAT0000646 | UUAAUGCUAAUCGUGAUAGGGGU | YP00204308 |
| hsa-miR-181a-5p | MIMAT0000256 | AACAUUCAACGCUGUCGGUGAGU | YP00206081 |
| hsa-miR-192-5p | MIMAT0000222 | CUGACCUAUGAAUUGACAGCC | YP00204099 |
| hsa-miR-199a-5p | MIMAT0000231 | CCCAGUGUUCAGACUACCUGUUC | YP00204494 |
| hsa-miR-214-3p | MIMAT0000271 | ACAGCAGGCACAGACAGGCAGU | YP00204510 |
| hsa-miR-221-3p | MIMAT0000278 | AGCUACAUUGUCUGCUGGGUUUC | YP00204532 |
| hsa-miR-222-3p | MIMAT0000279 | AGCUACAUCUGGCUACUGGGU | YP00204551 |
| hsa-miR-223-3p | MIMAT0000280 | UGUCAGUUUGUCAAAUACCCCA | YP00205986 |
| hsa-miR-323a-3p | MIMAT0000755 | CACAUUACACGGUCGACCUCU | YP00204278 |
| hsa-miR-451a | MIMAT0001631 | AAACCGUUACCAUUACUGAGUU | YP02119305 |
| hsa-miR-486-5p | MIMAT0002177 | UCCUGUACUGAGCUGCCCCGAG | YP00204001 |
| hsa-miR-491-5p | MIMAT0002807 | AGUGGGGAACCCUUCCAUGAGG | YP00204695 |
| hsa-miR-495-3p | MIMAT0002817 | AAACAAACAUGGUGCACUUCUU | YP00206015 |
| hsa-miR-499a-5p | MIMAT0002870 | UUAAGACUUGCAGUGAUGUUU | YP00205935 |
| hsa-miR-574-5p | MIMAT0004795 | UGAGUGUGUGUGUGUGAGUGUGU | YP02116206 |
|  |  |  |  |
|  |  |  |  |
|  |  |  |  |

| **Table S2.** Causes for ICU admission in non-COVID-19 patients. | |
| --- | --- |
|  | |
| Pneumonia | 5 (35.7) |
| Brain edema | 2 (14.3) |
| Hemoptysis | 1 (7.14) |
| Cervical abscess (surgical intervention) | 1 (7.14) |
| Severe myasthenia | 1 (7.14) |
| Laryngeal neoplasia | 1 (7.14) |
| Acute peritonitis | 1 (7.14) |
| Cardiorespiratory arrest | 1 (7.14) |
| Cardiogenic shock | 1 (7.14) |
|  |  |
| Data are shown as n (%). | |

| **Table S3**. miRNA ratios for Study Population 1 (COVID-19 vs. non-COVID-19) adjusted for age and sex. | | | |
| --- | --- | --- | --- |
|  |  |  |  |
| **miRNA ratio** | **Fold Change** | **p-value** | **AUC** |
|  |  |  |  |
| miR-122-5p/miR-199a-5p | 0.521 | 0.073 | 0.732 |
| miR-125a-5p/miR-133a-3p | 2.310 | 0.080 | 0.754 |
| miR-155-5p/miR-486-5p | 5.448 | 0.012 | 0.710 |
| miR-214-3p/miR-222-3p | 0.229 | 0.016 | 0.817 |
| miR-221-3p/miR-27a-3p | 1.695 | 0.051 | 0.730 |
|  |  |  |  |
|  |  |  |  |

**Table S4.** KEGG analysis for the miRNA ratios that showed statistical differences between COVID-19 and non-COVID-19 patients.

|  |  |  |  |
| --- | --- | --- | --- |
|  |  |  |  |
| **KEGG pathway** | **p-value** | **#genes** | **#miRNAs** |
|  |  |  |  |
| Proteoglycans in cancer | 4.56 x 10^-13^ | 107 | 9 |
| TGF-beta signaling pathway | 4.73 x 10^-10^ | 50 | 9 |
| Prion diseases | 3.57 x 10^-8^ | 16 | 7 |
| Lysine degradation | 1.56 x 10^-7^ | 28 | 9 |
| Hippo signaling pathway | 6.49 x 10^-7^ | 72 | 9 |
| Chronic myeloid leukemia | 1.36 x 10^-6^ | 47 | 9 |
| Viral carcinogenesis | 1.68 x 10^-6^ | 96 | 9 |
| AMPK signaling pathway | 2.27 x 10^-6^ | 72 | 9 |
| Hepatitis B | 2.86 x 10^-6^ | 74 | 9 |
| Colorectal cancer | 2.86 x 10^-6^ | 40 | 9 |
| ECM-receptor interaction | 8.86 x 10^-6^ | 36 | 9 |
| Glioma | 8.99 x 10^-6^ | 38 | 9 |
| Bacterial invasion of epithelial cells | 1.54 x 10^-5^ | 46 | 9 |
| Cell cycle | 1.73 x 10^-5^ | 68 | 9 |
| Adherens junction | 1.73 x 10^-5^ | 45 | 9 |
| Pancreatic cancer | 1.73 x 10^-5^ | 42 | 9 |
| Protein processing in endoplasmic reticulum | 3.14 x 10^-5^ | 87 | 9 |
| Non-small cell lung cancer | 3.40 x 10^-5^ | 34 | 9 |
| FoxO signaling pathway | 4.28 x 10^-5^ | 73 | 9 |
| Signaling pathways regulating pluripotency of stem cells | 4.28 x 10^-5^ | 71 | 9 |
| Other types of O-glycan biosynthesis | 1.02 x 10^-4^ | 16 | 7 |
| Endometrial cancer | 1.09 x 10^-4^ | 33 | 9 |
| Prostate cancer | 1.14 x 10^-4^ | 53 | 9 |
| Neurotrophin signaling pathway | 1.35 x 10^-4^ | 65 | 9 |
| Central carbon metabolism in cancer | 1.48 x 10^-4^ | 38 | 9 |
| Pathways in cancer | 1.48 x 10^-4^ | 174 | 9 |
| Transcriptional misregulation in cancer | 1.48 x 10^-4^ | 80 | 9 |
| Fatty acid biosynthesis | 1.62 x 10^-4^ | 4 | 5 |
| HIF-1 signaling pathway | 3.56 x 10^-4^ | 58 | 9 |
| Fatty acid elongation | 3.59 x 10^-4^ | 11 | 6 |
| Focal adhesion | 3.94 x 10^-4^ | 102 | 9 |
| mTOR signaling pathway | 6.21 x 10^-4^ | 37 | 9 |
| Fatty acid metabolism | 8.89 x 10^-4^ | 19 | 9 |
| Renal cell carcinoma | 8.89 x 10^-4^ | 40 | 9 |
| Sphingolipid signaling pathway | 9.37 x 10^-4^ | 59 | 9 |
| p53 signaling pathway | 9.37 x 10^-4^ | 41 | 9 |
| ErbB signaling pathway | 9.72 x 10^-4^ | 46 | 9 |

| **Table S5.** GO analysis for the miRNA ratios that showed statistical differences between COVID-19 and non-COVID-19 patients. | | | |
| --- | --- | --- | --- |
|  |  |  |  |
| **GO Category** | **p-value** | **#genes** | **#miRNAs** |
|  |  |  |  |
| Cellular nitrogen compound metabolic process | 4.51 x 10^-220^ | 1774 | 9 |
| Biosynthetic process | 2.72 x 10^-147^ | 1459 | 9 |
| Gene expression | 5.80 x 10^-123^ | 339 | 9 |
| Cellular protein modification process | 3.04 x 10^-103^ | 893 | 9 |
| Symbiosis encompassing mutualism through parasitism | 3.14 x 10^-76^ | 269 | 9 |
| Catabolic process | 3.14 x 10^-76^ | 731 | 9 |
| Small molecule metabolic process | 1.67 x 10^-73^ | 819 | 9 |
| Viral process | 5.37 x 10^-72^ | 241 | 9 |
| Biological process | 7.37 x 10^-68^ | 4641 | 9 |
| Cellular protein metabolic process | 3.09 x 10^-59^ | 222 | 9 |
| Mitotic cell cycle | 4.04 x 10^-56^ | 198 | 9 |
| Response to stress | 3.68 x 10^-46^ | 755 | 9 |
| Cellular component assembly | 3.70 x 10^-46^ | 476 | 9 |
| Nucleobase-containing compound catabolic process | 1.46 x 10^-45^ | 355 | 9 |
| Membrane organization | 1.69 x 10^-42^ | 251 | 9 |
| Neurotrophin TRK receptor signaling pathway | 4.07 x 10^-42^ | 125 | 9 |
| Macromolecular complex assembly | 6.12 x 10^-37^ | 331 | 9 |
| Cell death | 9.06 x 10^-36^ | 347 | 9 |
| Blood coagulation | 6.85 x 10^-32^ | 181 | 9 |
| Post-translational protein modification | 1.65 x 10^-28^ | 85 | 9 |
| Fc-epsilon receptor signaling pathway | 1.32 x 10^-27^ | 77 | 9 |
| mRNA metabolic process | 2.48 x 10^-26^ | 99 | 9 |
| DNA metabolic process | 4.67 x 10^-25^ | 280 | 9 |
| RNA metabolic process | 6.08 x 10^-25^ | 110 | 9 |
| Protein complex assembly | 3.18 x 10^-23^ | 268 | 9 |
| Cellular lipid metabolic process | 3.36 x 10^-23^ | 74 | 9 |
| Epidermal growth factor receptor signaling pathway | 1.85 x 10^-18^ | 91 | 9 |
| Chromatin organization | 2.09 x 10^-17^ | 68 | 9 |
| Platelet activation | 5.11 x 10^-17^ | 85 | 9 |
| Fc-gamma receptor signaling pathway involved in phagocytosis | 5.75 x 10^-17^ | 40 | 9 |
| G2/M transition of mitotic cell cycle | 1.10 x 10^-15^ | 72 | 8 |
| Transcription DNA-templated | 1.69 x 10^-15^ | 744 | 9 |
| TRIF-dependent toll-like receptor signaling pathway | 2.22 x 10^-15^ | 38 | 9 |
| Toll-like receptor 10 signaling pathway | 1.49 x 10^-14^ | 34 | 9 |
| Toll-like receptor TLR1:TLR2 signaling pathway | 2.70 x 10^-14^ | 35 | 9 |
| Toll-like receptor TLR6:TLR2 signaling pathway | 2.70 x 10^-14^ | 35 | 9 |
| Protein N-linked glycosylation via asparagine | 5.02 x 10^-14^ | 50 | 9 |
| Transcription initiation from RNA polymerase II promoter | 9.61 x 10^-14^ | 92 | 9 |
| Mitotic nuclear envelope disassembly | 1.31 x 10^-14^ | 25 | 8 |
| Intrinsic apoptotic signaling pathway | 1.75 x 10^-13^ | 42 | 9 |
| Myd88-independent toll-like receptor signaling pathway | 2.30 x 10^-13^ | 38 | 9 |
| Fibroblast growth factor receptor signaling pathway | 6.10 x 10^-13^ | 79 | 9 |
| Immune system process | 8.99 x 10^-13^ | 451 | 9 |
| Cell junction organization | 1.30 x 10^-12^ | 67 | 9 |
| Toll-like receptor 9 signaling pathway | 1.60 x 10^-12^ | 36 | 9 |
| Toll-like receptor 5 signaling pathway | 2.01 x 10^-12^ | 34 | 9 |
| Toll-like receptor 3 signaling pathway | 6.16 x 10^-12^ | 39 | 9 |
| Cellular component disassembly involved in execution phase of apoptosis | 2.53 x 10^-11^ | 26 | 9 |
| Vesicle-mediated transport | 3.46 x 10^-11^ | 316 | 9 |
| Protein targeting | 7.15 x 10^-11^ | 108 | 9 |
| Extracellular matrix organization | 9.11 x 10^-11^ | 130 | 9 |
| Toll-like receptor 4 signaling pathway | 1.14 x 10^-10^ | 43 | 9 |
| Platelet degranulation | 1.32 x 10^-10^ | 35 | 8 |
| Insulin receptor signaling pathway | 1.79 x 10^-10^ | 71 | 9 |
| Extracellular matrix disassembly | 3.28 x 10^-10^ | 46 | 9 |
| Toll-like receptor signaling pathway | 4.73 x 10^-10^ | 47 | 9 |
| Hexose transport | 4.97 x 10^-10^ | 23 | 8 |
| Regulation of glucose transport | 5.70 x 10^-10^ | 21 | 8 |
| Nuclear-transcribed mrna catabolic process deadenylation-dependent decay | 5.84 x 10^-10^ | 33 | 9 |
| Activation of signaling protein activity involved in unfolded protein response | 9.02 x 10^-10^ | 31 | 8 |
| Cell junction assembly | 9.02 x 10^-10^ | 31 | 9 |
| Toll-like receptor 2 signaling pathway | 9.02 x 10^-10^ | 35 | 9 |
| Cytoskeleton organization | 1.18 x 10^-9^ | 211 | 9 |
| Glycosaminoglycan metabolic process | 3.30 x 10^-9^ | 42 | 9 |
| Innate immune response | 3.31 x 10^-9^ | 214 | 9 |
| Stress-activated MAPK cascade | 5.26 x 10^-9^ | 28 | 9 |
| mRNA processing | 7.48 x 10^-9^ | 180 | 9 |
| Viral life cycle | 1.30 x 10^-8^ | 42 | 9 |
| RNA splicing | 1.30 x 10^-8^ | 118 | 9 |
| Phosphatidylinositol-mediated signaling | 1.39 x 10^-8^ | 54 | 9 |
| Protein maturation | 1.53 x 10^-8^ | 76 | 9 |
| Nucleocytoplasmic transport | 1.76 x 10^-8^ | 123 | 9 |
| Positive regulation of protein insertion into mitochondrial membrane involved in apoptotic signaling pathway | 2.61 x 10^-8^ | 19 | 9 |
| Termination of RNA polymerase II transcription | 3.45 x 10^-8^ | 24 | 8 |
| Apoptotic signaling pathway | 6.24 x 10^-8^ | 54 | 9 |
| Leukocyte migration | 7.60 x 10^-8^ | 46 | 9 |
| Cell motility | 9.40 x 10^-8^ | 170 | 9 |
| mRNA 3'-end processing | 1.07 x 10^-7^ | 22 | 8 |
| Transforming growth factor beta receptor signaling pathway | 1.50 x 10^-7^ | 74 | 9 |
| Cell cycle | 2.32 x 10^-7^ | 282 | 9 |
| Ribonucleoprotein complex assembly | 2.33 x 10^-7^ | 57 | 9 |
| Myd88-dependent toll-like receptor signaling pathway | 2.42 x 10^-7^ | 38 | 9 |
| Generation of precursor metabolites and energy | 2.55 x 10^-7^ | 102 | 9 |
| Phospholipid metabolic process | 2.20 x 10^-6^ | 58 | 9 |
| Post-Golgi vesicle-mediated transport | 2.32 x 10^-6^ | 24 | 9 |
| G1/S transition of mitotic cell cycle | 2.98 x 10^-6^ | 65 | 9 |
| Cytokine-mediated signaling pathway | 4.19 x 10^-6^ | 101 | 9 |
| O-glycan processing | 5.72 x 10^-6^ | 23 | 8 |
| Water-soluble vitamin metabolic process | 6.09 x 10^-6^ | 26 | 8 |
| Phosphatidylinositol biosynthetic process | 6.09 x 10^-6^ | 28 | 8 |
| Vitamin metabolic process | 6.09 x 10^-5^ | 28 | 8 |
| Transcription from RNA polymerase II promoter | 1.02 x 10^-5^ | 191 | 9 |
| Cell proliferation | 1.12 x 10^-5^ | 193 | 9 |
| Cellular component movement | 1.40 x 10^-5^ | 41 | 9 |
| SRP-dependent cotranslational protein targeting to membrane | 1.87 x 10^-5^ | 43 | 9 |
| Glycerophospholipid biosynthetic process | 2.01 x 10^-5^ | 31 | 8 |
| Carbohydrate metabolic process | 2.16 x 10^-5^ | 275 | 9 |
| Viral transcription | 2.77 x 10^-5^ | 28 | 9 |
| Chondroitin sulfate metabolic process | 4.58 x 10^-5^ | 20 | 8 |
| JAK-STAT cascade involved in growth hormone signaling pathway | 5.41 x 10^-5^ | 14 | 7 |
| Nucleobase-containing small molecule metabolic process | 6.43 x 10^-5^ | 24 | 8 |
| Nuclear-transcribed mRNA catabolic process nonsense-mediated decay | 8.55 x 10^-5^ | 46 | 9 |
| Nuclear-transcribed mRNA poly(A) tail shortening | 1.11 x 10^-4^ | 19 | 8 |
| Regulation of ubiquitin-protein ligase activity involved in mitotic cell cycle | 1.58 x 10^-4^ | 25 | 8 |
| Axon guidance | 1.68 x 10^-4^ | 133 | 9 |
| Sulfur compound metabolic process | 1.85 x 10^-4^ | 80 | 9 |
| Cell cycle arrest | 1.96 x 10^-4^ | 65 | 9 |
| Translational termination | 2.81 x 10^-4^ | 32 | 9 |
| Ras protein signal transduction | 6.91 x 10^-4^ | 40 | 9 |
| Transport | 7.17 x 10^-4^ | 1026 | 9 |
| Cofactor metabolic process | 8.71 x 10^-4^ | 70 | 9 |
| tRNA metabolic process | 9.16 x 10^-4^ | 54 | 8 |
| Positive regulation of viral transcription | 9.88 x 10^-4^ | 19 | 8 |
|  | | | |

| **Table S6.** Clinical characteristics of patients under IMV and patients with BAS sample available for microRNA quantification | | | | | |
| --- | --- | --- | --- | --- | --- |
|  | **ALL** | **Patients assisted by IMV** | **Patients with BAS sample available** | **p-value** | **Available data** |
|  | N=108 | N=51 | N=57 |  |  |
| **Sociodemographic characteristics** | | | | | |
| Age (years), median [P25; P75] | 62.0 [55.0;68.2] | 61.0 [52.5;67.0] | 63.0 [59.0;70.0] | 0.069 | 108 |
| Male, n (%) | 80 (74.1) | 34 (66.7) | 46 (80.7) | 0.149 | 108 |
| Smoking history, n (%) | | | | 0.707 | 95 |
| Former | 42 (44.2) | 18 (40.9) | 24 (47.1) |  | |
| Nonsmoker | 45 (47.4) | 23 (52.3) | 22 (43.1) |  |  |
| Current | 8 (8.42) | 3 (6.82) | 5 (9.80) |  |  |
| Alcoholism, n (%) | | | | 0.514 | 99 |
| Former | 2 (2.02) | 0 (0.00) | 2 (3.70) |  | |
| Non-alcoholic | 91 (91.9) | 43 (95.6) | 48 (88.9) |  |  |
| Current | 6 (6.06) | 2 (4.44) | 4 (7.41) |  |  |
| **Comorbidities** | | | | | |
| Hypertension, n (%) | 62 (57.4) | 28 (54.9) | 34 (59.6) | 0.762 | 108 |
| Type II Diabetes Mellitus, n (%) | 35 (32.4) | 13 (25.5) | 22 (38.6) | 0.212 | 108 |
| Obesity, n (%) | 61 (56.5) | 28 (54.9) | 33 (57.9) | 0.905 | 108 |
| Cardiovascular disease, n (%) | 16 (14.8) | 6 (11.8) | 10 (17.5) | 0.567 | 108 |
| COPD, n (%) | 9 (8.33) | 4 (7.84) | 5 (8.77) | 1.000 | 108 |
| Asthma, n (%) | 5 (4.63) | 2 (3.92) | 3 (5.26) | 1.000 | 108 |
| Chronic kidney disease, n (%) | 6 (5.56) | 4 (7.84) | 2 (3.51) | 0.418 | 108 |
| Chronic liver disease, n (%) | 4 (3.70) | 1 (1.96) | 3 (5.26) | 0.620 | 108 |
| Autoimmune disease, n (%) | 3 (2.78) | 2 (3.92) | 1 (1.75) | 0.601 | 108 |
| **ICU admission** | | | | | |
| Time since first symptoms to hospital admission (days), median [P25; P75] | 7.00 [4.00;8.00] | 7.00 [4.50;8.00] | 7.00 [4.00;8.00] | 0.958 | 108 |
| Time since first symptoms to ICU admission (days), median [P25; P75] | 8.00 [6.00;10.0] | 8.00 [6.00;10.0] | 8.00 [6.00;10.0] | 0.745 | 108 |
| Time since hospital admission to ICU admission (days), median [P25; P75] | 1.00 [0.00;3.00] | 1.00 [0.00;2.50] | 0.00 [0.00;3.00] | 0.974 | 108 |
| Oxygen saturation (%), median [P25; P75] | 94.0 [90.0;96.3] | 94.0 [90.0;97.0] | 94.0 [89.8;95.9] | 0.649 | 107 |
| FiO_2_ (%), median [P25; P75] | 70.0 [50.0;90.0] | 70.0 [42.5;94.5] | 70.0 [50.0;90.0] | 0.945 | 107 |
| PaO_2_ (mmHg), median [P25; P75] | 67.0 [51.0;87.0] | 67.0 [50.5;99.5] | 69.0 [52.5;83.8] | 0.626 | 85 |
| PaCO_2_ (mmHg), median [P25; P75] | 36.0 [32.0;42.0] | 36.0 [32.5;42.5] | 37.0 [32.0;41.0] | 0.895 | 85 |
| PaO_2_/FiO_2_, median [P25; P75] | 116 [79.0;163] | 132 [81.4;171] | 108 [78.3;156] | 0.450 | 85 |
| SaO_2_/FiO_2_, median [P25; P75] | 136 [103;190] | 134 [99.9;220] | 139 [105;172] | 0.950 | 107 |
| Glucose (mg/dL), median [P25; P75] | 143 [114;210] | 132 [114;195] | 160 [121;228] | 0.167 | 107 |
| Creatinine (mg/dL), median [P25; P75] | 0.84 [0.69;1.11] | 0.85 [0.67;1.10] | 0.84 [0.69;1.13] | 0.687 | 108 |
| C-reactive protein (mg/L), median [P25; P75] | 172 [85.9;234] | 199 [144;254] | 152 [66.0;197] | 0.006 | 105 |
| D-dimer (ng/mL), median [P25; P75] | 399 [285;675] | 428 [334;601] | 393 [285;728] | 0.501 | 93 |
| Leukocyte count (x10^9^/L), median [P25; P75] | 7.96 [6.34;11.1] | 8.27 [6.31;11.7] | 7.67 [6.37;10.2] | 0.544 | 108 |
| Neutrophil count (x10^9^/L) , median [P25; P75] | 6.96 [4.97;9.46] | 7.45 [5.14;10.2] | 6.43 [5.00;8.45] | 0.297 | 108 |
| Lymphocyte count (x10^9^/L) , median [P25; P75] | 0.66 [0.51;1.02] | 0.66 [0.48;1.02] | 0.65 [0.51;1.02] | 0.768 | 108 |
| Monocyte count (x10^9^/L), median [P25; P75] | 0.32 [0.18;0.47] | 0.29 [0.17;0.46] | 0.32 [0.20;0.47] | 0.825 | 108 |
| Platelet count (x10^9^/L), mean (SD) | 220 [176;270] | 225 [175;289] | 219 [176;245] | 0.201 | 107 |
| AST (U/L) , median [P25; P75] | 56.0 [35.0;80.0] | 51.5 [34.2;65.0] | 60.0 [38.0;89.0] | 0.130 | 73 |
| ALT (U/L) , median [P25; P75] | 37.0 [26.0;52.0] | 37.0 [25.0;47.8] | 37.0 [27.0;59.0] | 0.573 | 73 |
| Urea (mg/dL), median [P25; P75] | 48.5 [32.2;62.0] | 41.0 [28.0;62.0] | 51.0 [40.0;62.0] | 0.099 | 106 |
| APACHE-II score, median [P25; P75] | 16.0 [13.0;20.0] | 18.5 [14.2;21.8] | 16.0 [13.0;20.0] | 0.268 | 71 |
| **ICU Stay** | | | | | |
| ICU stay (days), median [P25; P75] | 20.0 [11.8;36.2] | 15.0 [8.00;21.5] | 32.0 [16.0;48.0] | <0.001 | 108 |
| Invasive mechanical ventilation duration (days), median [P25; P75] | 17.0 [8.00;30.5] | 11.0 [7.00;17.0] | 27.0 [14.0;40.0] | <0.001 | 106 |
| Prone positioning, n (%), mean (SD) | 87 (80.6) | 36 (70.6) | 51 (89.5) | 0.026 | 108 |
| Prone positioning duration (hours), median [P25; P75] | 44.5 [26.2;110] | 39.5 [23.2;71.2] | 62.0 [30.5;136] | 0.006 | 82 |
| Antibiotics, n (%) | 104 (96.3) | 50 (98.0) | 54 (94.7) | 0.620 | 108 |
| Hydroxychloroquine, n (%) | 45 (41.7) | 37 (72.5) | 8 (14.0) | <0.001 | 108 |
| Tocilizumab, n (%) | 65 (60.2) | 21 (41.2) | 44 (77.2) | <0.001 | 108 |
| Corticoids, n (%) | 101 (94.4) | 46 (90.2) | 55 (98.2) | 0.101 | 107 |
| **Initiation of invasive mechanical ventilation** | | | | | |
| Time since first symptoms to IMV (days), median [P25; P75] | 9.00 [7.00;11.8] | 9.00 [7.00;10.0] | 10.0 [7.00;13.0] | 0.150 | 106 |
| Time since hospital admission to IMV, (days), median [P25; P75] | 2.00 [0.00;4.75] | 2.00 [0.00;4.00] | 2.00 [0.00;6.00] | 0.298 | 106 |
| Time since ICU admission to IMV, (days), median [P25; P75] | 1.00 [0.00;2.00] | 0.00 [0.00;1.00] | 1.00 [0.00;3.00] | 0.057 | 106 |
| Oxygen saturation (%), median [P25; P75] | 96.0 [93.0;98.0] | 97.0 [93.0;98.0] | 95.3 [92.6;98.0] | 0.294 | 105 |
| FiO_2_ (%), median [P25; P75] | 70.0 [50.0;90.0] | 70.0 [60.0;94.0] | 70.0 [50.0;90.0] | 0.563 | 105 |
| PaO_2_ (mmHg), median [P25; P75] | 95.0 [66.0;120] | 95.0 [60.0;128] | 95.0 [70.0;115] | 0.919 | 77 |
| PaCO_2_ (mmHg), mean (SD) | 42.0 [36.0;51.0] | 39.0 [36.0;51.0] | 45.5 [38.0;50.5] | 0.225 | 77 |
| PaO_2_/FiO_2_, mean (SD) | 178 (87.0) | 147 (70.3) | 204 (92.3) | 0.042 | 36 |
| SaO_2_/FiO_2_, mean (SD) | 158 (47.1) | 139 (33.3) | 173 (51.5) | 0.021 | 36 |
| Glucose (mg/dL), median [P25; P75] | 138 [114;199] | 134 [113;191] | 146 [114;211] | 0.424 | 104 |
| Creatinine (mg/dL), median [P25; P75] | 0.81 [0.65;1.06] | 0.85 [0.65;1.09] | 0.78 [0.65;1.00] | 0.480 | 105 |
| C-reactive protein (mg/L), median [P25; P75] | 163 [44.2;240] | 210 [112;274] | 136 [19.9;210] | 0.004 | 103 |
| D-dimer (ng/mL) , median [P25; P75] | 565 [324;1330] | 527 [330;901] | 754 [296;2172] | 0.455 | 82 |
| Leukocyte count (x10^9^/L) , median [P25; P75] | 8.56 [6.41;12.1] | 9.39 [6.56;12.3] | 8.19 [6.29;11.6] | 0.494 | 105 |
| Neutrophil count (x10^9^/L) , median [P25; P75] | 7.50 [5.36;10.6] | 8.10 [5.62;11.2] | 7.30 [5.34;9.93] | 0.348 | 105 |
| Lymphocyte count (x10^9^/L) , median [P25; P75] | 0.76 [0.56;1.01] | 0.80 [0.52;1.01] | 0.70 [0.58;0.99] | 0.941 | 105 |
| Monocyte count (x10^9^/L) , median [P25; P75] | 0.29 [0.18;0.45] | 0.29 [0.18;0.43] | 0.31 [0.18;0.48] | 0.845 | 105 |
| Platelet count (x10^9^/L), mean (SD) | 234 [200;287] | 238 [205;299] | 232 [188;272] | 0.197 | 105 |
| AST (U/L) , median [P25; P75] | 53.5 [35.8;72.2] | 44.5 [34.2;64.2] | 60.0 [37.8;87.0] | 0.105 | 72 |
| ALT (U/L) , median [P25; P75] | 37.0 [26.0;52.2] | 37.0 [24.0;49.2] | 38.5 [27.5;55.5] | 0.710 | 72 |
| Urea (mg/dL) , median [P25; P75] | 46.0 [32.0;62.0] | 46.0 [28.0;60.0] | 49.5 [36.0;64.5] | 0.230 | 105 |

Continuous variables are expressed as the median [P25; P75] or mean (SD) and categorical variables are expressed as n (%). ALT: alanine aminotransferase; AST: aspartate aminotransferase; COPD: chronic obstructive pulmonary disease; FiO_2_: fraction of inspired oxygen; ICU: intensive care unit; IMV: invasive mechanical ventilation; LDH: lactate dehydrogenase; PaCO_2_: carbon dioxide partial pressure; PaO_2_: oxygen partial pressure; SaO_2_: oxygen saturation.

| **Table S7.** Clinical characteristics of patients with and without BAS sample available for microRNA quantification. | | | | | |
| --- | --- | --- | --- | --- | --- |
|  | **ALL** | **Patients without BAS sample available** | **Patients with BAS sample available** | **p-value** | **Available data** |
|  | N=77 | N=20 | N=57 |  |  |
| **Sociodemographic characteristics** | | | | | |
| Age (years), median [P25; P75] | 63.0 [57.0;69.0] | 63.5 [47.5;67.5] | 63.0 [59.0;70.0] | 0.320 | 77 |
| Male, n (%) | 63 (81.8) | 17 (85.0) | 46 (80.7) | 1.000 | 77 |
| Smoking history, n (%) | | | | 1.000 | 68 |
| Former | 32 (47.1) | 8 (47.1) | 24 (47.1) |  | |
| Nonsmoker | 30 (44.1) | 8 (47.1) | 22 (43.1) |  |  |
| Current | 6 (8.82) | 1 (5.88) | 5 (9.80) |  |  |
| Alcoholism, n (%) | | | | 1.000 | 72 |
| Former | 2 (2.78) | 0 (0.00) | 2 (3.70) |  | |
| Non-alcoholic | 65 (90.3) | 17 (94.4) | 48 (88.9) |  |  |
| Current | 5 (6.94) | 1 (5.56) | 4 (7.41) |  |  |
| **Comorbidities** | | | | | |
| Hypertension, n (%) | 44 (57.1) | 10 (50.0) | 34 (59.6) | 0.626 | 77 |
| Type II Diabetes Mellitus, n (%) | 27 (35.1) | 5 (25.0) | 22 (38.6) | 0.410 | 77 |
| Obesity, n (%) | 43 (55.8) | 10 (50.0) | 33 (57.9) | 0.726 | 77 |
| Cardiovascular disease, n (%) | 13 (16.9) | 3 (15.0) | 10 (17.5) | 1.000 | 77 |
| COPD, n (%) | 7 (9.09) | 2 (10.0) | 5 (8.77) | 1.000 | 77 |
| Asthma, n (%) | 5 (6.49) | 2 (10.0) | 3 (5.26) | 0.600 | 77 |
| Chronic kidney disease, n (%) | 3 (3.90) | 1 (5.00) | 2 (3.51) | 1.000 | 77 |
| Chronic liver disease, n (%) | 4 (5.19) | 1 (5.00) | 3 (5.26) | 1.000 | 77 |
| Autoimmune disease, n (%) | 2 (2.60) | 1 (5.00) | 1 (1.75) | 0.455 | 77 |
| **ICU admission** | | | | | |
| Time since first symptoms to hospital admission (days), median [P25; P75] | 7.00 [4.00;8.00] | 7.00 [4.75;8.00] | 7.00 [4.00;8.00] | 0.925 | 77 |
| Time since first symptoms to ICU admission (days), median [P25; P75] | 8.00 [6.00;10.0] | 8.00 [6.75;10.0] | 8.00 [6.00;10.0] | 0.888 | 77 |
| Time since hospital admission to ICU admission (days), median [P25; P75] | 1.00 [0.00;3.00] | 1.00 [0.00;3.00] | 0.00 [0.00;3.00] | 0.652 | 77 |
| Oxygen saturation (%), median [P25; P75] | 94.4 [89.8;96.0] | 95.8 [90.5;97.2] | 94.0 [89.8;95.9] | 0.240 | 76 |
| FiO_2_ (%), median [P25; P75] | 70.0 [50.0;90.0] | 75.0 [48.8;98.5] | 70.0 [50.0;90.0] | 0.380 | 76 |
| PaO_2_ (mmHg), median [P25; P75] | 70.5 [54.2;85.8] | 76.0 [63.8;101] | 69.0 [52.5;83.8] | 0.141 | 58 |
| PaCO_2_ (mmHg), median [P25; P75] | 36.5 [32.0;43.8] | 35.5 [30.5;48.8] | 37.0 [32.0;41.0] | 0.910 | 58 |
| PaO_2_/FiO_2_, median [P25; P75] | 108 [78.1;156] | 115 [77.0;148] | 108 [78.3;156] | 0.896 | 58 |
| SaO_2_/FiO_2_, median [P25; P75] | 136 [102;184] | 127 [97.5;185] | 139 [105;172] | 0.406 | 76 |
| Glucose (mg/dL), median [P25; P75] | 140 [114;213] | 120 [108;148] | 160 [121;228] | 0.018 | 77 |
| Creatinine (mg/dL), median [P25; P75] | 0.86 [0.69;1.07] | 0.90 [0.77;0.98] | 0.84 [0.69;1.13] | 0.926 | 77 |
| C-reactive protein (mg/L), mean (SD) | 159 (101) | 193 (112) | 148 (95.7) | 0.124 | 75 |
| D-dimer (ng/mL), median [P25; P75] | 400 [293;717] | 455 [372;594] | 393 [285;728] | 0.224 | 66 |
| Leukocyte count (x10^9^/L), median [P25; P75] | 7.67 [6.41;11.1] | 7.46 [6.53;11.8] | 7.67 [6.37;10.2] | 0.830 | 77 |
| Neutrophil count (x10^9^/L), median [P25; P75] | 6.43 [5.00;9.15] | 6.69 [5.42;10.4] | 6.43 [5.00;8.45] | 0.597 | 77 |
| Lymphocyte count (x10^9^/L), median [P25; P75] | 0.65 [0.51;1.06] | 0.69 [0.47;1.07] | 0.65 [0.51;1.02] | 0.785 | 77 |
| Monocyte count (x10^9^/L), median [P25; P75] | 0.31 [0.19;0.47] | 0.29 [0.18;0.47] | 0.32 [0.20;0.47] | 0.991 | 77 |
| Platelet count (x10^9^/L), mean (SD) | 219 [176;245] | 222 [186;248] | 219 [176;245] | 0.550 | 77 |
| AST (U/L), median [P25; P75] | 56.0 [39.0;86.0] | 54.0 [42.5;69.2] | 60.0 [38.0;89.0] | 0.770 | 53 |
| ALT (U/L), median [P25; P75] | 40.0 [28.0;58.0] | 42.0 [32.0;52.0] | 37.0 [27.0;59.0] | 0.558 | 53 |
| Urea (mg/dL), median [P25; P75] | 49.0 [34.0;62.0] | 37.5 [24.0;63.0] | 51.0 [40.0;62.0] | 0.093 | 77 |
| APACHE-II score, median [P25; P75] | 16.0 [13.2;20.0] | 18.0 [16.0;19.0] | 16.0 [13.0;20.0] | 0.260 | 62 |
| **ICU Stay** | | | | | |
| ICU stay (days), median [P25; P75] | 27.0 [14.0;43.0] | 16.0 [11.8;22.0] | 32.0 [16.0;48.0] | 0.001 | 77 |
| Invasive mechanical ventilation duration (days), median [P25; P75] | 23.0 [11.0;34.0] | 13.0 [10.8;17.2] | 27.0 [14.0;40.0] | 0.001 | 77 |
| Prone positioning, n (%), mean (SD) | 66 (85.7) | 15 (75.0) | 51 (89.5) | 0.141 | 77 |
| Prone positioning duration (hours), median [P25; P75] | 60.0 [30.0;126] | 48.0 [28.5;72.5] | 62.0 [30.5;136] | 0.156 | 63 |
| Antibiotics, n (%) | 74 (96.1) | 20 (100) | 54 (94.7) | 0.564 | 77 |
| Hydroxychloroquine, n (%) | 24 (31.2) | 16 (80.0) | 8 (14.0) | <0.001 | 77 |
| Tocilizumab, n (%) | 54 (70.1) | 10 (50.0) | 44 (77.2) | 0.045 | 77 |
| Corticoids, n (%) | 74 (97.4) | 19 (95.0) | 55 (98.2) | 0.460 | 76 |
| **Initiation of invasive mechanical ventilation** | | | | | |
| Time since first symptoms to IMV (days), median [P25; P75] | 10.0 [7.00;12.0] | 9.00 [7.75;11.0] | 10.0 [7.00;13.0] | 0.366 | 77 |
| Time since hospital admission to IMV, (days), median [P25; P75] | 2.00 [0.00;5.00] | 2.00 [0.75;3.25] | 2.00 [0.00;6.00] | 0.559 | 77 |
| Time since ICU admission to IMV, (days), median [P25; P75] | 1.00 [0.00;2.00] | 0.00 [0.00;1.00] | 1.00 [0.00;3.00] | 0.060 | 77 |
| Oxygen saturation (%), median [P25; P75] | 96.0 [93.8;98.2] | 98.0 [96.0;98.8] | 95.3 [92.6;98.0] | 0.078 | 76 |
| FiO_2_ (%), median [P25; P75] | 70.0 [50.0;91.2] | 70.0 [49.5;100] | 70.0 [50.0;90.0] | 0.807 | 76 |
| PaO_2_ (mmHg), median [P25; P75] | 99.9 (39.0) | 108 (40.3) | 96.3 (38.4) | 0.308 | 57 |
| PaCO_2_ (mmHg), mean (SD) | 44.8 (11.7) | 42.8 (10.7) | 45.7 (12.1) | 0.372 | 57 |
| PaO_2_/FiO_2_, mean (SD) | 202 (85.7) | 195 (69.1) | 204 (92.3) | 0.803 | 27 |
| SaO_2_/FiO_2_, mean (SD) | 166 (50.2) | 146 (43.6) | 173 (51.5) | 0.204 | 27 |
| Glucose (mg/dL), median [P25; P75] | 136 [111;190] | 121 [97.0;151] | 146 [114;211] | 0.073 | 75 |
| Creatinine (mg/dL), median [P25; P75] | 0.82 [0.65;1.00] | 0.91 [0.70;1.01] | 0.78 [0.65;1.00] | 0.376 | 76 |
| C-reactive protein (mg/L), median [P25; P75] | 158 [31.4;235] | 231 [141;281] | 136 [19.9;210] | 0.003 | 74 |
| D-dimer (ng/mL) , median [P25; P75] | 589 [324;1884] | 479 [336;1042] | 754 [296;2172] | 0.726 | 58 |
| Leukocyte count (x10^9^/L), median [P25; P75] | 8.38 [6.42;12.1] | 8.98 [6.67;12.5] | 8.19 [6.29;11.6] | 0.454 | 76 |
| Neutrophil count (x10^9^/L), median [P25; P75] | 7.46 [5.46;10.3] | 7.95 [5.63;11.7] | 7.30 [5.34;9.93] | 0.396 | 76 |
| Lymphocyte count (x10^9^/L), median [P25; P75] | 0.72 [0.57;1.07] | 0.84 [0.56;1.07] | 0.70 [0.58;0.99] | 0.777 | 76 |
| Monocyte count (x10^9^/L), median [P25; P75] | 0.30 [0.18;0.47] | 0.28 [0.18;0.44] | 0.31 [0.18;0.48] | 0.995 | 76 |
| Platelet count (x10^9^/L), mean (SD) | 232 [199;272] | 234 [212;272] | 232 [188;272] | 0.555 | 76 |
| AST (U/L, median [P25; P75] | 56.0 [36.5;84.5] | 56.0 [33.0;68.5] | 60.0 [37.8;87.0] | 0.577 | 51 |
| ALT (U/L), median [P25; P75] | 40.0 [27.0;60.5] | 44.0 [27.0;77.0] | 38.5 [27.5;55.5] | 0.549 | 51 |
| Urea (mg/dL), median [P25; P75] | 46.5 [33.0;64.5] | 43.0 [24.0;57.8] | 49.5 [36.0;64.5] | 0.211 | 76 |

Continuous variables are expressed as the median [P25; P75] or mean (SD) and categorical variables are expressed as n (%). ALT: alanine aminotransferase; AST: aspartate aminotransferase; COPD: chronic obstructive pulmonary disease; FiO_2_: fraction of inspired oxygen; ICU: intensive care unit; IMV: invasive mechanical ventilation; LDH: lactate dehydrogenase; PaCO_2_: carbon dioxide partial pressure; PaO_2_: oxygen partial pressure; SaO_2_: oxygen saturation.

| **Table S8.** Microbiological and fungal examinations of bronchial aspirate samples. | | | | | |
| --- | --- | --- | --- | --- | --- |
|  |  |  |  |  |  |
|  | **All** | **Survivor** | **Non-survivor** | **p-value** | **n** |
|  |  |  |  |  |  |
| Microbiological examination |  |  |  |  | 49 |
| *Enterobacter aerogenes* | 1 (2.04) | 1 (2.94) | 0 (0.00) | 1.000 |  |
| *Haemophilus influenzae* | 2 (4.08) | 2 (5.88) | 0 (0.00) | 1.000 |  |
| *Neisseria meningitidis* and *Streptococcus pneumoniae* | 1 (2.04) | 1 (2.94) | 0 (0.00) | 1.000 |  |
| *Serratia Marcescens* | 3 (6.12) | 3 (8.82) | 0 (0.00) | 0.543 |  |
| *Staphylococcus aureus* | 11 (22.4) | 7 (20.6) | 4 (26.7) | 0.716 |  |
| *Flora Saprofita* | 12 (24.5) | 6 (17.6) | 6 (40.0) | 0.148 |  |
| *Legionella* (negative) | 48 (100) | 34 (100) | 14 (100) | - | 48 |
| Fungal examination | | | |  | 39 |
| *Aspergillus terreus* | 1 (2.56) | 0 (0.00) | 1 (9.09) | 0.282 |  |
| *Candida albicans* | 3 (7.69) | 3 (10.7) | 0 (0.00) | 0.545 |  |
| *Candida dubliniensis* | 1 (2.56) | 0 (0.00) | 1 (9.09) | 0.282 |  |
| *Candida parapsilosis* | 1 (2.56) | 1 (3.57) | 0 (0.00) | 1.000 |  |
| *Candida tropicalis* | 1 (2.56) | 0 (0.00) | 1 (9.09) | 0.282 |  |
| *Nocardia sp* (negative) | 49 (100) | 34 (100) | 15 (100) | 1.000 | 49 |
|  |  |  |  |  |  |
| Data are shown as n (%) | | | | | |

| **Table S9.** miRNA ratios for Study Population 2 (survivor vs nonsurvivor) adjusted for age and sex. | | | |
| --- | --- | --- | --- |
|  |  |  |  |
| **miRNA ratio** | **Fold Change** | **p-value** | **AUC** |
|  |  |  |  |
| miR-1-3p/miR-124-3p | 1.880 | 0.068 | 0.692 |
| miR-125b-5p/miR-34a-5p | 1.274 | 0.114 | 0.679 |
| miR-126-3p/miR-16-5p | 1.870 | 0.021 | 0.681 |
| miR-199a-5p/miR-9-5p | 3.150 | 0.001 | 0.802 |
| miR-221-3p/miR-491-5p | 0.650 | 0.055 | 0.704 |
|  |  |  |  |

| **Table S10.** KEGG analysis for the miRNA ratios that showed statistical differences between survivors and nonsurvivors to ICU stay. | | | |
| --- | --- | --- | --- |
|  |  |  |  |
| **KEGG pathway** | **p-value** | **#genes** | **#miRNAs** |
|  |  |  |  |
| Proteoglycans in cancer | 1.66 x 10^-12^ | 134 | 9 |
| Fatty acid metabolism | 2.71 x 10^-12^ | 30 | 7 |
| Cell cycle | 1.13 x 10^-9^ | 91 | 9 |
| Adherens junction | 1.13 x 10^-9^ | 57 | 9 |
| Pathways in cancer | 1.78 x 10^-9^ | 245 | 9 |
| Hippo signaling pathway | 5.68 x 10^-7^ | 94 | 9 |
| Fatty acid biosynthesis | 3.99 x 10^-6^ | 7 | 6 |
| Hepatitis B | 7.87 x 10^-6^ | 92 | 9 |
| Prostate cancer | 7.87 x 10^-6^ | 66 | 9 |
| Fatty acid elongation | 9.16 x 10^-6^ | 16 | 6 |
| Other types of O-glycan biosynthesis | 1.38 x 10^-5^ | 21 | 7 |
| Prion diseases | 2.86 x 10^-5^ | 19 | 8 |
| Colorectal cancer | 8.67 x 10^-5^ | 46 | 9 |
| Small cell lung cancer | 1.29 x 10^-4^ | 62 | 9 |
| p53 signaling pathway | 1.49 x 10^-4^ | 52 | 9 |
| TGF-beta signaling pathway | 1.64 x 10^-4^ | 52 | 9 |
| Chronic myeloid leukemia | 1.64 x 10^-4^ | 53 | 9 |
| Glioma | 2.20 x 10^-4^ | 44 | 9 |
| Glycosaminoglycan biosynthesis - chondroitin sulfate / dermatan sulfate | 4.58 x 10^-4^ | 14 | 5 |
| Bacterial invasion of epithelial cells | 4.58 x 10^-4^ | 53 | 9 |
| Pancreatic cancer | 4.58 x 10^-4^ | 49 | 9 |
| Acute myeloid leukemia | 4.58 x 10^-4^ | 41 | 9 |
| Protein processing in endoplasmic reticulum | 6.30 x 10^-4^ | 106 | 9 |
| Transcriptional misregulation in cancer | 7.04 x 10^-4^ | 111 | 9 |
|  |  |  |  |

| **Table S11.** GO analysis for the miRNA ratios that showed statistical differences between survivors and nonsurvivors to ICU stay. | | | |
| --- | --- | --- | --- |
|  |  |  |  |
| **GO Category** | **p-value** | **#genes** | **#miRNAs** |
|  |  |  |  |
| Cellular nitrogen compound metabolic process | 1.77 x 10^-183^ | 2311 | 9 |
| Gene expression | 2.77 x 10^-126^ | 419 | 9 |
| Biosynthetic process | 7.89 x 10^-117^ | 1903 | 9 |
| Symbiosis encompassing mutualism through parasitism | 7.93 x 10^-90^ | 357 | 9 |
| Small molecule metabolic process | 1.56 x 10^-89^ | 1170 | 9 |
| Viral process | 2.31 x 10^-87^ | 322 | 9 |
| Cellular protein modification process | 1.34 x 10^-81^ | 1141 | 9 |
| Cellular protein metabolic process | 7.24 x 10^-79^ | 309 | 9 |
| Catabolic process | 1.26 x 10^-72^ | 979 | 9 |
| Neurotrophin TRK receptor signaling pathway | 6.25 x 10^-60^ | 176 | 9 |
| Mitotic cell cycle | 1.62 x 10^-58^ | 251 | 9 |
| Membrane organization | 1.03 x 10^-48^ | 341 | 9 |
| Nucleobase-containing compound catabolic process | 2.32 x 10^-46^ | 475 | 9 |
| Cellular component assembly | 3.57 x 10^-46^ | 646 | 9 |
| Macromolecular complex assembly | 8.46 x 10^-40^ | 453 | 9 |
| Blood coagulation | 1.68 x 10^-38^ | 249 | 9 |
| Response to stress | 3.18 x 10^-36^ | 1010 | 9 |
| Fc-epsilon receptor signaling pathway | 2.75 x 10^-34^ | 102 | 9 |
| Post-translational protein modification | 1.09 x 10^-33^ | 111 | 9 |
| Cell death | 6.60 x 10^-30^ | 450 | 9 |
| mRNA metabolic process | 1.94 x 10^-29^ | 129 | 9 |
| RNA metabolic process | 5.06 x 10^-25^ | 140 | 9 |
| Epidermal growth factor receptor signaling pathway | 1.42 x 10^-24^ | 128 | 9 |
| Protein complex assembly | 2.57 x 10^-24^ | 369 | 9 |
| Toll-like receptor 10 signaling pathway | 2.65 x 10^-23^ | 49 | 9 |
| TRIF-dependent toll-like receptor signaling pathway | 1.91 x 10^-22^ | 53 | 9 |
| Toll-like receptor TLR1:TLR2 signaling pathway | 3.42 x 10^-22^ | 50 | 9 |
| Toll-like receptor TLR6:TLR2 signaling pathway | 3.42 x 10^-22^ | 50 | 9 |
| DNA metabolic process | 1.20 x 10^-21^ | 369 | 9 |
| Myd88-independent toll-like receptor signaling pathway | 7.08 x 10^-20^ | 54 | 9 |
| Toll-like receptor 5 signaling pathway | 3.09 x 10^-19^ | 49 | 9 |
| Activation of signaling protein activity involved in unfolded protein response | 1.49 x 10^-18^ | 49 | 9 |
| Viral life cycle | 2.50 x 10^-18^ | 70 | 9 |
| Chromatin organization | 6.75 x 10^-18^ | 86 | 9 |
| Cellular lipid metabolic process | 2.72 x 10^-17^ | 82 | 9 |
| Protein N-linked glycosylation via asparagine | 2.73 x 10^-17^ | 67 | 9 |
| Toll-like receptor 3 signaling pathway | 4.71 x 10^-17^ | 55 | 9 |
| Toll-like receptor 9 signaling pathway | 9.96 x 10^-17^ | 49 | 9 |
| Platelet activation | 1.19 x 10^-16^ | 108 | 9 |
| Cellular component disassembly involved in execution phase of apoptosis | 1.30 x 10^-16^ | 36 | 9 |
| Nucleobase-containing small molecule metabolic process | 4.76 x 10^-15^ | 46 | 8 |
| Toll-like receptor 4 signaling pathway | 5.09 x 10^-15^ | 61 | 9 |
| Glycosaminoglycan metabolic process | 7.81 x 10^-15^ | 63 | 9 |
| Water-soluble vitamin metabolic process | 8.37 x 10^-15^ | 46 | 9 |
| Toll-like receptor signaling pathway | 1.16 x 10^-14^ | 68 | 9 |
| Fibroblast growth factor receptor signaling pathway | 2.21 x 10^-14^ | 106 | 9 |
| Cell junction assembly | 2.93 x 10^-14^ | 44 | 9 |
| Toll-like receptor 2 signaling pathway | 3.07 x 10^-14^ | 50 | 9 |
| Mitotic nuclear envelope disassembly | 7.34 x 10^-14^ | 29 | 7 |
| Vitamin metabolic process | 1.12 x 10^-13^ | 48 | 9 |
| Cell junction organization | 1.61 x 10^-13^ | 88 | 9 |
| Stress-activated MAPK cascade | 1.93 x 10^-13^ | 40 | 9 |
| Fc-gamma receptor signaling pathway involved in phagocytosis | 1.96 x 10^-12^ | 42 | 9 |
| Viral transcription | 3.50 x 10^-12^ | 48 | 9 |
| Sulfur compound metabolic process | 3.54 x 10^-12^ | 139 | 9 |
| Protein targeting | 1.01 x 10^-11^ | 148 | 9 |
| Transcription initiation from RNA polymerase II promoter | 2.87 x 10^-11^ | 114 | 9 |
| Immune system process | 7.46 x 10^-11^ | 634 | 9 |
| Post-Golgi vesicle-mediated transport | 1.72 x 10^-10^ | 36 | 8 |
| Cofactor metabolic process | 3.64 x 10^-10^ | 121 | 9 |
| Phosphatidylinositol-mediated signaling | 1.17 x 10^-9^ | 73 | 9 |
| Extracellular matrix organization | 1.22 x 10^-9^ | 173 | 9 |
| Myd88-dependent toll-like receptor signaling pathway | 1.93 x 10^-9^ | 53 | 9 |
| G2/M transition of mitotic cell cycle | 1.97 x 10^-9^ | 78 | 8 |
| Platelet degranulation | 2.73 x 10^-9^ | 41 | 9 |
| Extracellular matrix disassembly | 4.77 x 10^-9^ | 56 | 9 |
| Hexose transport | 5.48 x 10^-9^ | 26 | 7 |
| Positive regulation of type I interferon production | 1.13 x 10^-8^ | 40 | 9 |
| Ribonucleoprotein complex assembly | 1.18 x 10^-8^ | 79 | 9 |
| SRP-dependent cotranslational protein targeting to membrane | 1.20 x 10^-8^ | 65 | 9 |
| Translational termination | 1.48 x 10^-8^ | 52 | 9 |
| Regulation of glucose transport | 1.70 x 10^-8^ | 23 | 7 |
| Nuclear-transcribed mRNA catabolic process nonsense-mediated decay | 4.46 x 10^-8^ | 71 | 9 |
| Glycerophospholipid biosynthetic process | 8.52 x 10^-8^ | 45 | 9 |
| Chondroitin sulfate metabolic process | 1.23 x 10^-7^ | 29 | 8 |
| Innate immune response | 1.34 x 10^-7^ | 293 | 9 |
| Nucleotide-binding domain leucine rich repeat containing receptor signaling pathway | 2.16 x 10^-7^ | 25 | 9 |
| Termination of RNA polymerase II transcription | 2.32 x 10^-7^ | 28 | 8 |
| Vesicle-mediated transport | 2.85 x 10^-7^ | 424 | 9 |
| Phospholipid metabolic process | 3.45 x 10^-7^ | 80 | 9 |
| Intrinsic apoptotic signaling pathway | 3.50 x 10^-7^ | 41 | 9 |
| Cellular component movement | 3.71 x 10^-7^ | 58 | 9 |
| Protein maturation | 4.56 x 10^-7^ | 97 | 9 |
| Positive regulation of protein insertion into mitochondrial membrane involved in apoptotic signaling pathway | 4.61 x 10^-7^ | 21 | 9 |
| mRNA 3'-end processing | 1.61 x 10^-6^ | 25 | 8 |
| Leukocyte migration | 2.47 x 10^-6^ | 56 | 9 |
| Long-chain fatty acyl-CoA biosynthetic process | 2.93 x 10^-6^ | 14 | 6 |
| C-terminal protein lipidation | 3.10 x 10^-6^ | 20 | 6 |
| Transforming growth factor beta receptor signaling pathway | 3.56 x 10^-6^ | 95 | 9 |
| Cell motility | 4.58 x 10^-6^ | 230 | 9 |
| G1/S transition of mitotic cell cycle | 4.91 x 10^-6^ | 87 | 9 |
| DNA strand elongation involved in DNA replication | 9.44 x 10^-6^ | 20 | 6 |
| Insulin receptor signaling pathway | 1.36 x 10^-5^ | 79 | 9 |
| Biological process | 1.40 x 10^-5^ | 6575 | 9 |
| Nuclear-transcribed mRNA catabolic process nonsense-mediated decay | 1.56 x 10^-5^ | 33 | 8 |
| Regulation of ubiquitin-protein ligase activity involved in mitotic cell cycle | 2.05 x 10^-5^ | 34 | 8 |
| Energy reserve metabolic process | 2.21 x 10^-5^ | 45 | 9 |
| Generation of precursor metabolites and energy | 2.26 x 10^-5^ | 132 | 9 |
| Nucleobase-containing small molecule interconversion | 3.14 x 10^-5^ | 13 | 7 |
| Regulation of defense response to virus by virus | 3.81 x 10^-5^ | 17 | 8 |
| tRNA metabolic process | 5.11 x 10^-5^ | 79 | 9 |
| Cell cycle | 6.41 x 10^-5^ | 387 | 9 |
| Sulfur amino acid metabolic process | 6.56 x 10^-5^ | 17 | 7 |
| Nucleotide-binding oligomerization domain containing signaling pathway | 7.75 x 10^-5^ | 15 | 8 |
| Homeostatic process | 1.09 x 10^-4^ | 313 | 9 |
| Preassembly of GPI anchor in ER membrane | 1.29 x 10^-4^ | 12 | 5 |
| 'De novo' posttranslational protein folding | 1.40 x 10^-4^ | 23 | 6 |
| Cytoskeleton organization | 1.53 x 10^-4^ | 266 | 9 |
| Anatomical structure morphogenesis | 1.80 x 10^-4^ | 50 | 7 |
| Adherens junction organization | 2.10 x 10^-4^ | 26 | 8 |
| CENP-A containing nucleosome assembly | 2.17 x 10^-4^ | 24 | 7 |
| Dolichol-linked oligosaccharide biosynthetic process | 2.17 x 10^-4^ | 25 | 8 |
| Cellular amino acid metabolic process | 2.28 x 10^-4^ | 171 | 9 |
| Positive regulation of ubiquitin-protein ligase activity involved in mitotic cell cycle | 2.30 x 10^-4^ | 31 | 8 |
| Phosphatidylinositol biosynthetic process | 2.74 x 10^-4^ | 32 | 8 |
| Antigen processing and presentation of exogenous peptide antigen via MHC class II | 3.69 x 10^-4^ | 53 | 9 |
| Endoplasmic reticulum unfolded protein response | 3.69 x 10^-4^ | 62 | 9 |
| Nucleocytoplasmic transport | 3.69 x 10^-4^ | 149 | 9 |
| Anaphase-promoting complex-dependent proteasomal ubiquitin-dependent protein catabolic process | 6.86 x 10^-4^ | 38 | 8 |
| Regulation of transcription from RNA polymerase II promoter in response to hypoxia | 6.92 x 10^-4^ | 17 | 7 |
| In utero embryonic development | 7.85 x 10^-4^ | 157 | 9 |
| Axon guidance | 7.91 x 10^-4^ | 185 | 9 |

**SUPPLEMENTAL FIGURES**

**Figure S1**


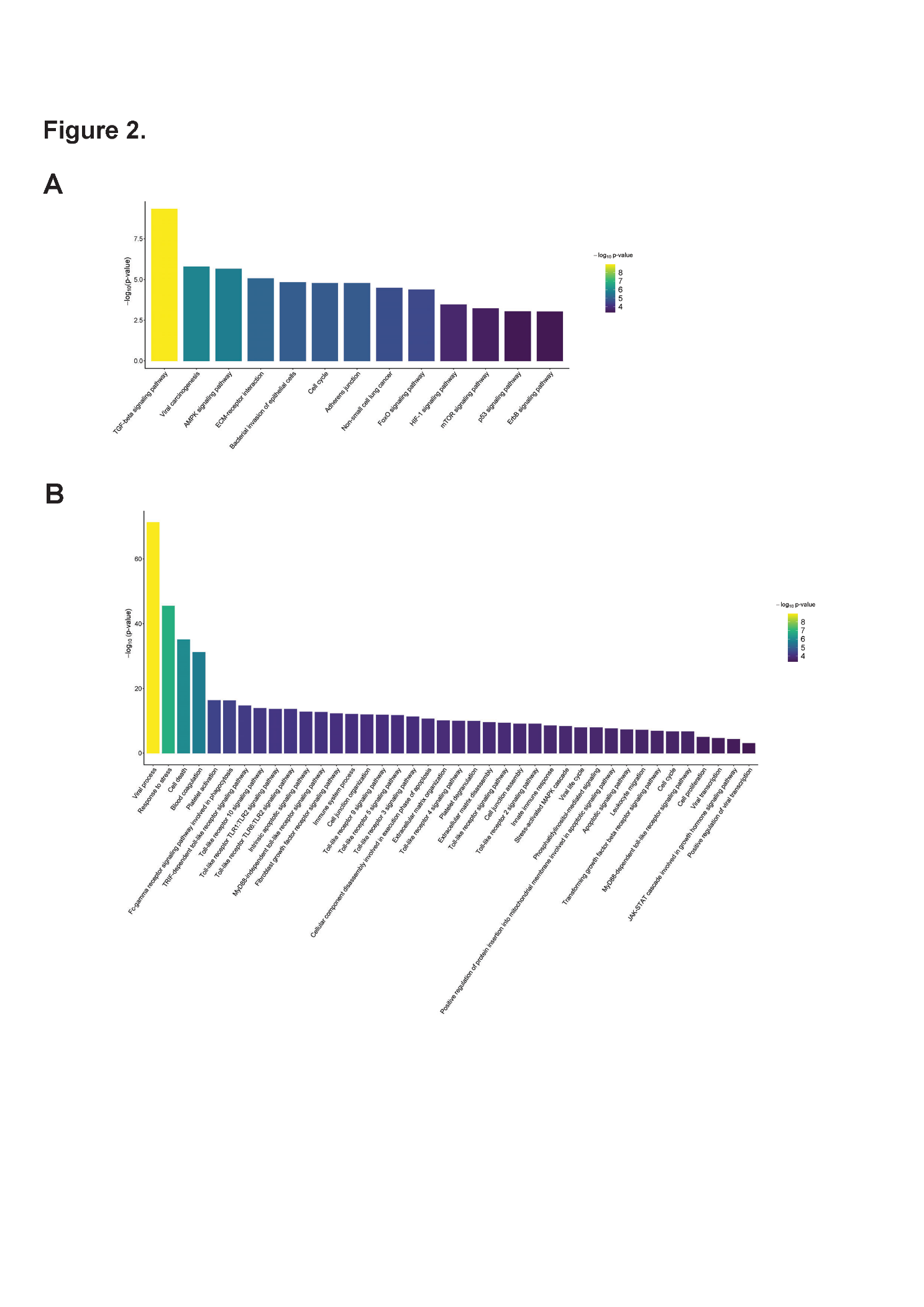


**Figure S1. KEGG pathway (A) and Gene Ontology (B) analyses for critically ill COVID-19 and non-COVID-19 patients (selected for COVID-19-related pathogenesis).** The microRNA ratios that showed differences between critically ill COVID-19 and non-COVID-19 patients were included in the analyses. The p-value denotes the significance of the biological process. Pathways and biological processes related to COVID-19 pathophysiology are displayed. The false discovery rate (FDR)-adjusted p-value cutoff was 0.001.

**Figure S2**


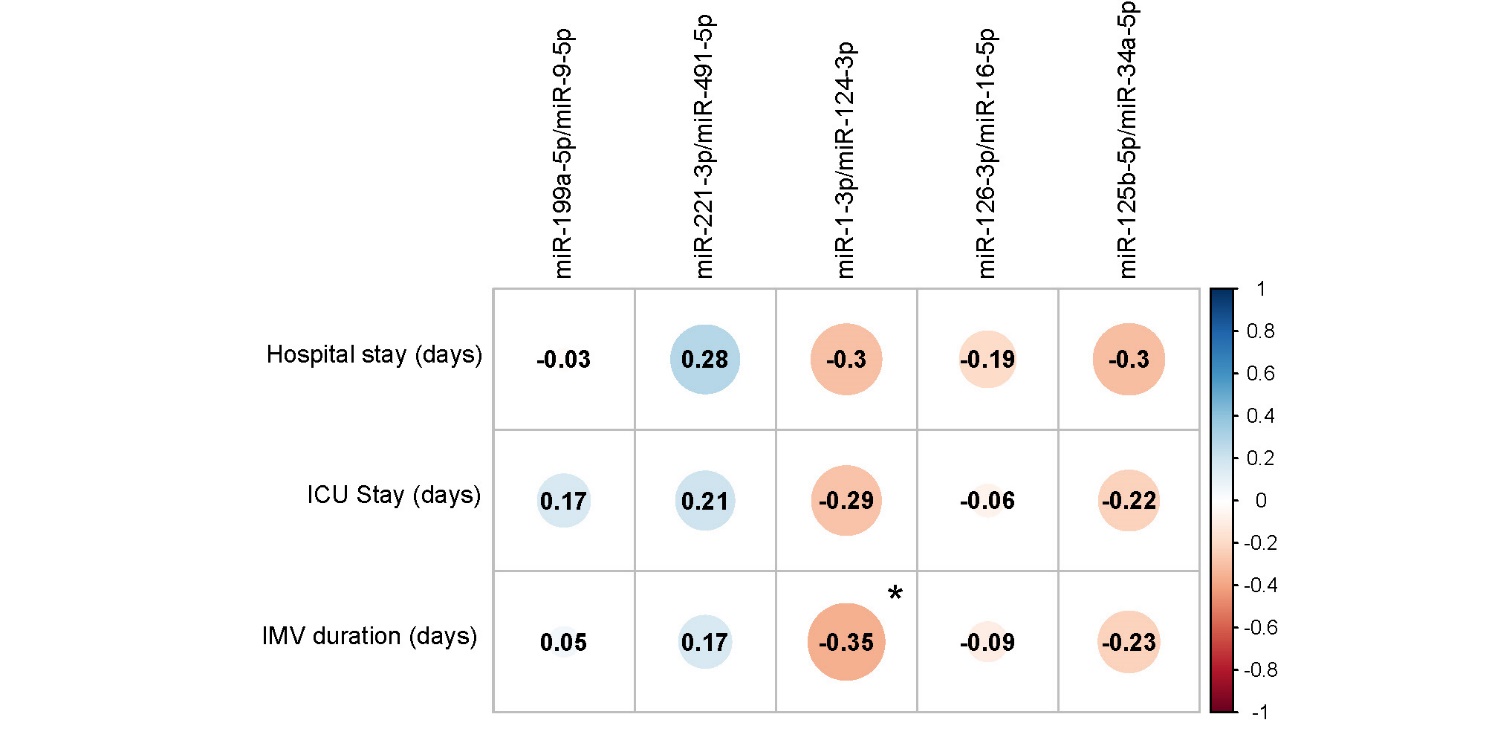


**Figure S2. Correlations between miRNA ratios and duration of IMV, hospital stay and ICU stay.** ICU: Intensive Care Unit, IMV: Invasive mechanical ventilation. *: p-value<0.05

**Figure S3**


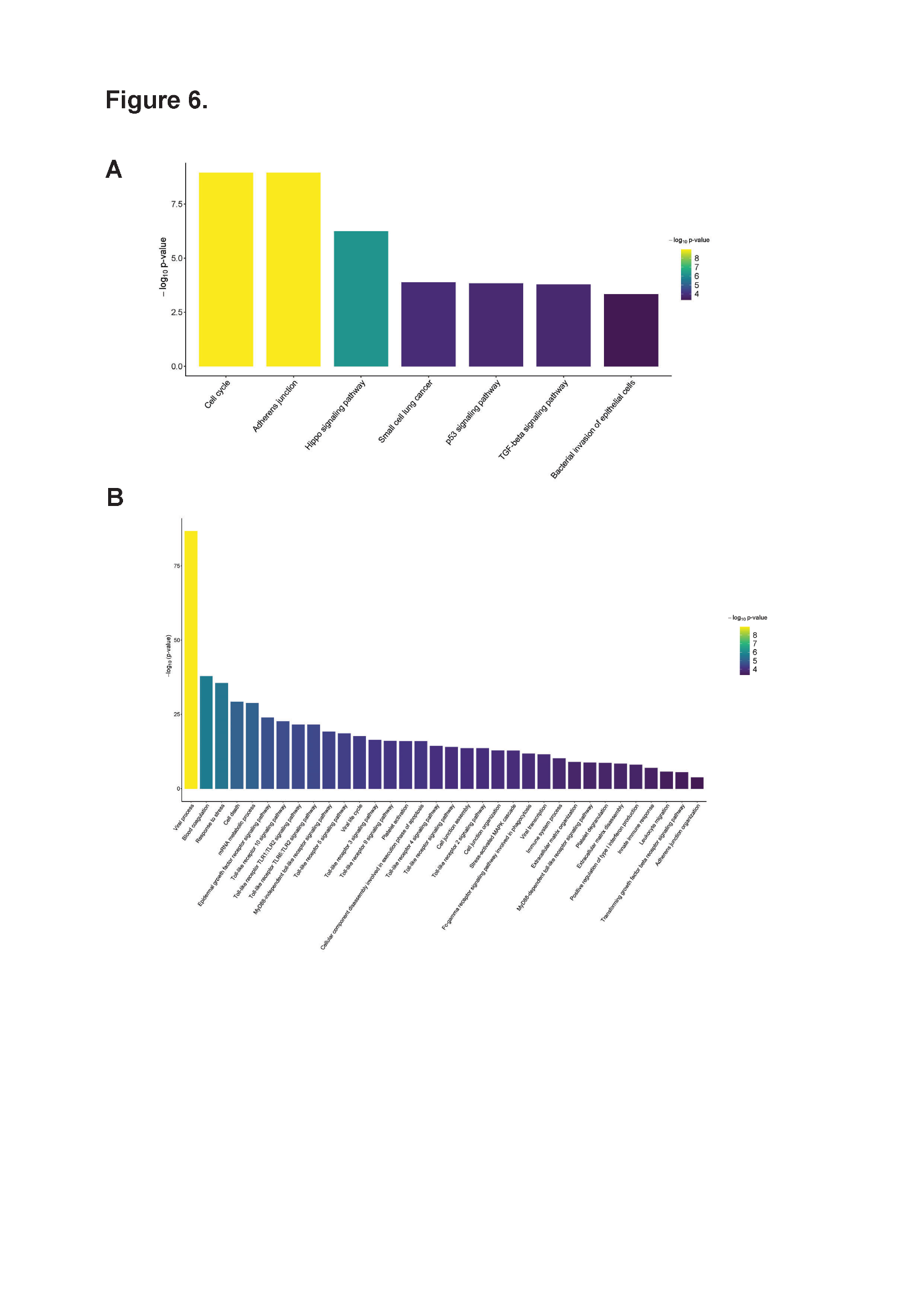


**Figure S3. KEGG pathway (A) and Gene Ontology (B) analyses of COVID-19 survivors and nonsurvivors to ICU stay (selected for COVID-19-related pathogenesis).** The microRNA ratios that showed differences between survivors and nonsurvivors to ICU stay were included in the analyses. The p-value denotes the significance of the biological process. Pathways and biological processes related to COVID-19 pathophysiology are displayed. The false discovery rate (FDR)-adjusted p-value cutoff was 0.001.
